# Functional Genomics Insights Into the Pathogenicity, Habitat Fitness, and Mechanisms Modifying Plant Development of *Rhodococcus* sp. PBTS1 and PBTS2

**DOI:** 10.3389/fmicb.2020.00014

**Published:** 2020-01-30

**Authors:** Danny Vereecke, Yucheng Zhang, Isolde M. Francis, Paul Q. Lambert, Jolien Venneman, Rio A. Stamler, James Kilcrease, Jennifer J. Randall

**Affiliations:** ^1^Entomology, Plant Pathology, and Weed Science, New Mexico State University, Las Cruces, NM, United States; ^2^Department of Plant Pathology, University of Florida, Gainesville, FL, United States; ^3^Department of Biology, California State University, Bakersfield, CA, United States; ^4^Department of Plants and Crops, Ghent University, Ghent, Belgium

**Keywords:** actinobacteria, virulence loss, niche partitioning, plant hormones, auxin, cytokinin, ethylene, volatiles

## Abstract

Pistachio Bushy Top Syndrome (PBTS) is a recently emerged disease that has strongly impacted the pistachio industry in California, Arizona, and New Mexico. The disease is caused by two bacteria, designated PBTS1 that is related to *Rhodococcus corynebacterioides* and PBTS2 that belongs to the species *R. fascians*. Here, we assessed the pathogenic character of the causative agents and examined their chromosomal sequences to predict the presence of particular functions that might contribute to the observed co-occurrence and their effect on plant hosts. In diverse assays, we confirmed the pathogenicity of the strains on “UCB-1” pistachio rootstock and showed that they can also impact the development of tobacco species, but concurrently inconsistencies in the ability to induce symptoms were revealed. We additionally evidence that *fas* genes are present only in a subpopulation of pure PBTS1 and PBTS2 cultures after growth on synthetic media, that these genes are easily lost upon cultivation in rich media, and that they are enriched for in an *in planta* environment. Analysis of the chromosomal sequences indicated that PBTS1 and PBTS2 might have complementary activities that would support niche partitioning. Growth experiments showed that the nutrient utilization pattern of both PBTS bacteria was not identical, thus avoiding co-inhabitant competition. PBTS2 appeared to have the potential to positively affect the habitat fitness of PBTS1 by improving its resistance against increased concentrations of copper and penicillins. Finally, mining the chromosomes of PBTS1 and PBTS2 suggested that the bacteria could produce cytokinins, auxins, and plant growth-stimulating volatiles and that PBTS2 might interfere with ethylene levels, in support of their impact on plant development. Subsequent experimentation supported these *in silico* predictions. Altogether, our data provide an explanation for the observed pathogenic behavior and unveil part of the strategies used by PBTS1 and PBTS2 to interact with plants.

## Introduction

The cosmopolitan Actinobacteria, one of the largest phyla within the Bacteria, are acclaimed for their metabolic versatility and their capacity to produce biologically active agents with applications in medicine, agriculture, and biotechnology (Barka et al., 2016; Lee et al., 2018). Within the Actinobacteria, members of the genus *Rhodococcus* have been studied extensively because of their extraordinary ability to degrade a wide spectrum of xenobiotic and organic compounds, targeting them for bioremediation. Additionally, their often large genomes (up to 10 Mb), encode highly specialized pathways that are extremely useful for biotransformation and biocatalysis purposes (Ceniceros et al., 2017; Elsayed et al., 2017; Alvarez, 2019; Gupta et al., 2019). Nevertheless, besides these valuable characteristics, some *Rhodococcus* species use their metabolic capacities to infect animals, humans, and plants (von Bargen and Haas, 2009; Stes et al., 2011b, 2013; Anastasi et al., 2016).

Until recently, the capacity among rhodococci to interact with plants seemed to be restricted to the plant-pathogenic species *R. fascians* that is now considered to be part of the “*Rhodococcus fascians* assemblage” (Sangal et al., 2019). *R. fascians* isolates typically cause diverse developmental alterations in their hosts, including excessive shoot formation, leaf deformations, and stunted growth, collectively designated the leafy gall syndrome (Stes et al., 2011b). The host range of these bacteria encompasses a broad spectrum of mostly herbaceous dicotyledonous plants, although some woody plants and few monocotyledons are sensitive as well (Putnam and Miller, 2007; Depuydt et al., 2008b). Key to symptom development is the bacterial production of an array of cytokinins via gene products of the fasciation (*fas*) operon encoded on a linear virulence plasmid in most pathogenic isolates (Crespi et al., 1992; Pertry et al., 2009, 2010; Francis et al., 2012; Creason et al., 2014; Radhika et al., 2015; Jameson et al., 2019) and the concomitant modification of the hormone landscape of the infected plant (Depuydt et al., 2008a, 2009a,2009b; Stes et al., 2011a, 2012, 2015; Jameson et al., 2019). Additionally, the pathogenic model strain D188 possesses a chromosomal locus *vic* (virulence in chromosome) implicated in symptom persistence that encodes a malate synthase believed to be involved in the catabolism of leafy gall-specific nutrients (Vereecke et al., 2002). Despite the absolute requirement of the linear plasmid for virulence in most leafy gall inducers, an important contribution of co-option of chromosomal and plasmid-encoded functions seems to guarantee a successful interaction with plants (Creason et al., 2014; Francis et al., 2016). As a result of metagenomic studies in search of biostimulants, it has now become evident that diverse *Rhodococcus* species are established members of the plant microbiome (Francis and Vereecke, 2019). Additionally, a novel disease on “UCB-1” pistachio rootstock trees, designated Pistachio Bushy Top Syndrome (PBTS) has been associated with the presence of two *Rhodococcus* species that act synergistically on their host (Stamler et al., 2015a, b). So, *R. fascians* is no longer the sole plant interactor nor the unique plant pathogen within the *Rhodococcus* genus.

PBTS is characterized by an array of symptoms, including stunted growth, formation of additional shoots, and a disturbed root development. Furthermore, affected “UCB-1” plants exhibit a strongly reduced capacity to accept a *Pistacia vera* graft and in the unlikely event that grafting is successful, cracked gall-like tissue carrying ectopic shoots develops at the graft junctions with a weakened stock-scion union as a consequence. Altogether, these disease aspects prevent the plants from going into production (Stamler et al., 2015b). The causative agents of PBTS were designated *Rhodococcus* isolate 1 and *Rhodococcus* isolate 2 (Stamler et al., 2015b), but here will be referred to as PBTS1 and PBTS2, respectively. 16S rRNA gene sequence analysis of several PBTS1 isolates obtained from symptomatic “UCB-1” trees, showed that they cluster together with *R. corynebacterioides*. Although *R. corynebacterioides* strains have not been studied extensively, they have been reported as endophytes from the xylem of eggplant and the leaves of *Arabidopsis thaliana* (Traw et al., 2007; Achari and Ramesh, 2014), inhabitants of the phyllosphere of apples (He et al., 2012), and as efficient degraders of aflatoxin (formerly *Flavobacterium aurantiacum*; Teniola et al., 2005; Risa et al., 2018), oil (Bayat et al., 2015), and rubber products (Pan et al., 2009). They have also been described as the cause of various infections in patients (Al Akhrass et al., 2012; Kitamura et al., 2012; Vergidis et al., 2017; Khalil et al., 2019) and as cancer killers exhibiting potent anti-malignancy activity (Zhou et al., 2017). In contrast, PBTS2 isolates grouped together with *R. fascians* (Stamler et al., 2015b). In agreement with the similarities between the symptoms associated with the leafy gall and PBTS syndromes, in both PBTS1 and PBTS2, the presence was demonstrated by PCR of the *fasD* gene, encoding an isopentenyl transferase that mediates the rate-limiting step of cytokinin biosynthesis (Crespi et al., 1992), the *fasA* gene coding for a P450 monooxygenase involved in zeatin production (Pertry et al., 2010; Genbank accession MF278335 for PBTS2 *fasA*), and *vic* (Stamler et al., 2015b). Although the presence of *fasD* is 100% correlated with virulence in leafy gall inducers (e.g., Crespi et al., 1992; Stange et al., 1996; Nikolaeva et al., 2009; Creason et al., 2014), its role in the PBTS strains remains to be determined. Moreover, the synergistic activity of both species in symptom development and the absence of genuine leafy galls in the PBTS syndrome suggest that the strategies used by these bacteria differ from those of leafy gall inducers.

Unexpectedly, the *fas* genes could not be detected in the assembled draft genome sequences of either of the PBTS strains (Stamler et al., 2016; Randall et al., 2018) and as a consequence the pathogenic status of PBTS1 and PBTS2 has been questioned (Savory et al., 2017). Here, we addressed this controversy and used a functional genomics approach to find clues as to how the PBTS bacteria would co-occur and modulate plant development. First, we analyzed the features of the available draft genomic sequences of PBTS1 and PBTS2 and compared them to each other and to those of *R. fascians* D188 (Stamler et al., 2016). We reassessed the pathogenicity of the PBTS strains on “UCB-1” plants and on two *Nicotiana* species and tested the hypothesis that the *fas* genes in the PBTS strains are easily lost during sub-culturing in rich medium, possibly the reason for their absence from the genome sequences (Randall et al., 2018; Vereecke, 2018). Additionally, based on the consistent co-isolation of both species from “UCB-1” trees exhibiting PBTS symptoms (Stamler et al., 2015a, b), we postulated that both isolates would not compete with each other for resources and would possibly exhibit complementary activities that would allow them to grow well in each other’s presence which might be a prerequisite to synergize for optimal symptom formation (Stamler et al., 2015b). To this end, we mined the chromosomes of PBTS1 and PBTS2 to predict the occurrence of particular metabolic activities and phenotypes that might be important to thrive in their habitats, to associate with each other, and to modify plant development.

## Materials and Methods

### Bioinformatics

The accession numbers of the chromosome and plasmid sequences analyzed are: CP015219 (chromosome) for PBTS1, CP015220 (chromosome) and CP015221 (pD188-like plasmid) for PBTS2, and CP015235 (chromosome) and CP015237 (pD188) for D188 (Stamler et al., 2016). For the linear plasmid sequence of strain D188, pFiD188, we used the Sanger sequenced version JN093097 (Francis et al., 2016) and corrected sequencing ambiguities based on CP015236 (Stamler et al., 2016). The CRISPR loci of these genomes were identified by the CRISPRCasFinder server^1^ with default parameters (Couvin et al., 2018).

For the analysis of the genome similarities of the PBTS isolates and D188, chromosome and plasmid sequences were aligned by progressive MAUVE (Darling et al., 2004). For the comparison of the six PBTS isolates, D188, and *R. corynebacterioides* NBRC 14404, pair-wise Average Nucleotide Identity (ANI) values were calculated with nucmer^2^ and the *in silico* DNA–DNA hybridization (*is*DDH) values by means of the Genome-to-Genome Distance Calculator (GGDC). The genomic DNA sequences were uploaded into the GGDC 2.1 Web server^3^ (Meier-Kolthoff et al., 2013). The point estimates from Formula 2 were utilized as the *is*DDH estimates.

Orthologous groups of the three reference genomes were determined with OrthoMCL v. 1.4 with the default parameters (Li et al., 2003). First, the OrthoMCL program carried out reciprocal comparisons of each protein sequence with BLASTp, where after the generated reciprocal BLASTp *e*-values were used to create a matrix analyzed by a Markov cluster (MCL) algorithm. Based on this analysis, OrthoMCL detected orthologous and paralogous genes of the three genomes and clustered them into orthologous groups.

For functional annotation and metabolic pathway reconstruction, a Cluster of Orthologous Genes (COG) annotation was conducted by a BLASTp search (threshold e-value 10^–5^) against the COG myva database^4^. The Kyoto Encyclopedia of Genes and Genomes (KEGG) annotation was done with BlastKOALA^5^ (Kanehisa et al., 2016). Bacteria as target taxonomy group and the species_prokaryotes database were chosen in the scoring scheme for K number assignment. Proteins assigned with a valid K number were used to generate lists of reconstructed KEGG pathways with the KEGG Mapper^6^.

### Bacterial Strains and Growth Conditions

The bacterial strains used in this study were PBTS1 and PBTS2 (Stamler et al., 2015b, 2016), D188 (Desomer et al., 1988), and the PBTS isolates Rhodo 10, Rhodo 11, Rhodo 12, and Rhodo 13, which were obtained from “UCB-1” trees exhibiting PBTS symptoms during the outbreak reported by Stamler et al. (2015a, b).

The bacterial strains were grown at 23–28°C for 2 days on D2 (Kado and Heskett, 1970), yeast extract broth (YEB Broth, PhytoTechnology Laboratories, Overland Park, KS, United States), or nutrient broth (NB; Difco^TM^, ThermoFisher Scientific, Waltham, MA, United States) solidified with 1.5% agar (Difco^TM^ Agar Technical). For infections, the bacteria were scraped from D2 plates and suspended in water to an OD_600_ of 2.0, unless otherwise indicated.

### Pathogenicity Assays on “UCB-1” and *Nicotiana* Species

Unbudded clonal “UCB-1” rootstock trees in plugs, 8 weeks acclimatized from tissue culture, were purchased from different commercial suppliers. Shoot infections were done by Agdia Inc (Elkhart, Indiana, United States). A 1/1 mixture of the PBTS bacteria [10^7^ (colony-forming units) CFU/ml] resuspended in a buffer containing 10 mM MgCl_2_ and 10 mM 2-(N-morpholino)ethanesulfonic acid (MES) (pH 5.5) was applied on the leaves of 3 months old plants; control plants were mock-infected with water. The plants were placed in a growth chamber at 24°C with a 12 h light/12-dark regime and their development was followed over several months during which the plants grew well. Images were taken 12 months after inoculation. Dip infections were done in the greenhouse facilities of the Research Institute for Agriculture, Fisheries and Food (ILVO-Plant, Melle, Belgium). The PBTS bacteria grown on D2 plates were scraped and resuspended in a buffer containing 10 mM MgCl_2_ and 10 mM MES (pH 5.5) (OD_600_ of 0.3), and mixed in a 1:1 ratio. The aerial part of 25 plants, 5–10 cm in height, and the plugs were dipped in the bacterial suspension and planted immediately in 20 cm deep pots with a diameter of 16 cm (volume 3 l); the substrate consisted of 60% peat moss, 35% perlite, and 5% vermiculite. At the base of the stem, 10 ml of the suspension was poured. As controls, 10 plants were treated in the same way but buffer was used as mock inoculum. The plants were placed in a greenhouse and to increase humidity, the planted material was covered by plastic for 1 week. Plant development was followed over a period of 4.5 months.

The pathogenicity assays with “UCB-1” seedlings were done at CSU Bakersfield. Uncoated “UCB-1” seeds were soaked in sterile water with aeration for 24 h, where after the water was replaced with 1% bleach and the seeds were soaked for another 24 h. Subsequently, the seeds were surface sterilized with 70% ethanol for 5 min, 20% bleach for 5 min, and rinsed three times with sterile water. The sterilized seeds were placed between sterile wet paper towels in sealed sterile containers, 5 seeds per MK-5 container (Caisson Laboratories, Smithfield, UT, United States) and incubated at 30°C. From day 5 after the surface sterilization, the seeds were checked daily for germination, upon which they were placed right below the surface of moist sterile potting mixture. Plant growth was continued at 30°C in a plant growth chamber with a 16 h light/8 h dark photoperiod. Three weeks old seedlings were infected by placing 20 μl drops of bacterial suspensions (prepared as described above) on each leaflet and axil; water was used as mock-infected control. Symptom development was monitored over a period of 30 days. The experiment was repeated twice with three plants for each condition.

Pathogenicity assessment on *N. tabacum* W38 including the seedling infection assay and infection via dipping has been described previously (Maes et al., 2001). The former assay was repeated twice with 25 seedlings for each condition; the latter assay was done only once with three replicates of four plants for each condition.

For infections of *N. benthamiana*, seeds were first surface sterilized (agitation in 2% bleach for 2 min, in 70% ethanol for 2 min, and three rinses of 2 min in deionized water) and planted in sterile vermiculite. After germination, the plants were transplanted when approximately 5.5 cm tall into autoclaved LM-40 High Porosity Mix (Lambert, Rivière-Ouelle, Quebec, Canada) inoculated with 50 ml aqueous suspensions of either PBTS1 or PBTS2 cultures, grown in YEB and diluted to 10^7^ CFU/ml; uninoculated potting mix was used as negative control. For each condition, five replicates of single plants were used.

### Assessment of the Presence of *fas* and *vic* Genes

The primer sequences used for the detection of *vicA*, *vicA2*, 16S rRNA, ITS, *fasC*, *fasD*, *fasE*, and *fasF* either by quantitative (q)PCR or endpoint PCR are given in Supplementary Table S1 (Nikolaeva et al., 2012; Dhandapani et al., 2017; DeLorenzo and Moon, 2018).

qPCR was utilized to determine the amount of *fasD* DNA relative to DNA of chromosomal genes in PBTS1, PBTS2, and D188. PBTS1 has two homologs of the *vicA* gene that are amplified with different primer sets. In PBTS1, *vicA2* is the chromosomal homolog equivalent to *vicA* in PBTS2 and D188, whereas the *vicA* homolog in PBTS1 should be considered as a pathogenicity gene. Genomic DNA (gDNA) was extracted from bacterial pellets using the MoBio PowerLyser PowerSoil DNA isolation kit (QIAGEN, Carlsbad, United States). The PCR reactions were conducted in triplicate on a CFX92 real-time Thermal Cycler (Bio-Rad, Hercules, CA, United States) using iTaq^TM^ Universal SYBR^®^ Green Supermix (BioRad), 100 ng of gDNA as template, and 0.2 μM of each forward and reverse primer for the following target genes *vicA*, *vicA2* (PBTS1), and *fasD* (*fasD-JR* primers). Reaction conditions included an initial denaturation step for 3 min at 95°C, followed by 40 cycles of 10 s at 95°C and 30 s at 60°C. A final melting curve was done from 65°C until 95°C at 0.5 s increments of 1°C.

For a second independent, preliminary qPCR experiment, gDNA was extracted from three different 2 days old PBTS1-like and PBTS2-like isolates and from D188 cultures obtained after direct inoculation of glycerol stocks in 5 ml YEB. The 12 μl qPCR reaction mixture was made with GoTaq^®^ qPCR Master Mix (Promega, Madison, WI, United States) and consisted of 6.25 μl reaction buffer, 0.625 μl 5 μM forward (FW) primer, 0.625 μl 5 μM reversed (RV) primer, 0.208 μl dye, 3.3 μl water, and 1 μl gDNA (10 ng). The 16S rRNA gene was used as a reference and the target genes were *fasC* (using the *fasD-PJ* primers that do not target *fasD* as reported; Dhandapani et al., 2017), *fasE* and *fasF*. Single runs for each DNA sample were done on a CFX96^TM^ Real-Time PCR detection system (Bio-Rad) as follows: 10 min at 95°C, followed by 40 cycles of 20 s at 95°C, 20 s at 58°C and 20 s at 72°C. After 90 s at 72°C, the melt curve of the amplicons was determined by increasing the temperature with 1°C every 5 s from 72°C until 95°C. The amplicons were sent for sequencing to LGC (Berlin, Germany).

The presence of the *fasD* gene was additionally analyzed with endpoint PCR. PBTS1 and PBTS2 were inoculated from glycerol stocks in 5 ml YEB medium and after 2 days of growth at 28°C, a 1-ml sample was taken for gDNA extraction. The remainder of the cultures was diluted in 500 ml YEB and grown for another 2 days, where after another 1 ml sample was taken for gDNA extraction. The 25 μl PCR reaction mixtures were prepared as follows: 5 μl 5 × colorless GoTaq^®^ reaction buffer (Promega), 0.5 μl dNTP mix (10 mM stock; Promega), 1 μl FW primer (10 μM stock), 1 μl RV primer (10 μM stock), 0.125 μl GoTaq^®^ DNA Polymerase (Promega), 16 μl water, and 1 μl (10 ng) gDNA. As a reference the ITS region was used (Nikolaeva et al., 2009) and the target gene was amplified with the *fasD-DV* primers. gDNA from D188 was used as a positive control and a template-free reaction as a negative control. The PCR was run on a T100^TM^ Thermal Cycler (Bio-Rad) with the following settings: 2 min at 95°C, followed by 35 cycles of 20 s at 95°C, 20 s at 50°C and 1 min at 72°C, and a final extension of 5 min at 72°C. The *fasD* amplicons were sent for sequencing to LGC (Berlin, Germany).

Genomes of Rhodo 10, Rhodo 11, Rhodo 12, and Rhodo 13 were sequenced at Oxford Genomics Center (University of Oxford, United Kingdom) on the HiSeq 4000 platform (PE150 reads; Illumina, San Diego, CA, United States), yielding at least 72 Gb of data in total or 750 Mb on average per sample (depending on the genome size, GC content, and DNA quality). The library was prepared with an in-house adapted protocol of the New England Biolabs (Ipswich, MA, United States) prep kit.

### *In planta* Enrichment Experiment

Surface sterilized *N. benthamiana* seeds were directly added to pots with autoclaved LM-40 potting mix (Lambert) to allow germination. Three pots for each treatment were placed in the same glass irrigation dish and sub-irrigated for the duration of the experiment. After germination, plants were thinned to leave three plants per pot for a total of nine plants per treatment.

When *N. benthamiana* seedlings reached the peak of their cotyledon stage, PBTS1 and PBTS2 cultures were generated from glycerol stocks on solid nutrient agar (NA) medium and grown for 2 days. Two-μl aliquots of aqueous suspensions obtained from the plate cultures (OD_600_ set to 0.7) were pipetted onto each of the two emerged leaves of each plant and 2 ml samples were taken for gDNA extraction and qPCR analysis of *vic* and *fasD* as target genes as described above. After 3 weeks, the plants from each treatment were cut above the soil line, added to individual conical tubes containing 5 ml of Dulbecco’s phosphate-buffered saline, and shaken for 1 min. Then, 2.5 ml of the buffer was plated onto D2 medium to obtain epiphytic bacteria. Additionally, the plant tissues were surface sterilized with the seed-sterilization method described above, transferred to individual conical tubes with 3 ml deionized water, and ground into a green liquid, of which 500 μl was plated onto D2 medium to obtain endophytic bacteria. Individual bacteria from both sets of plates were grown on D2 medium for gDNA extraction, 300 ng of which was used for qPCR analysis of *vicA* and *fasD* as described above.

### Carbon Source Utilization, Penicillin and Copper Resistance, and Antagonism Tests

Bacterial growth assays on different carbohydrates and amino acids as carbon sources and the generation of antibiograms were done as described (Francis et al., 2016). In the latter experiment, PBTS1 titers in the halo’s were quantified by resuspending the bacteria present on a 5 mm diameter agar plug harvested just aside from the antibiotic-containing disc in 300 μl sterile water. Subsequent serial dilutions were plated on YEB and bacteria were counted 4 days later.

For the Cu resistance test, a plate diffusion assay was done as described (Hassen et al., 1998) with CuSO_4_.5H_2_O concentrations of 100, 150, and 200 mM. Prior to inoculation, bacterial titers were set at an OD_600_ of 0.1. Bacterial sensitivity was scored by measuring the length of the growth in the inoculation streak (in mm) after 6 days of incubation at 25°C. The occurrence of PBTS1 at the tip of the growth closest to the highest Cu concentration was evaluated with a stereomicroscope M165FC equipped with a DFC 310 FX color camera (Leica, Wetzlar, Germany).

For the antagonism test, liquid YEB cultures of PBTS1, PBTS2, D188 and *Bacillus subtilis* were grown for 2 days under gentle agitation at 28°C. Fifty-μl samples (OD_600_ of 1.0) were diluted in 5 ml of YEB medium with 0.5% agar cooled to 45°C. This mixture was poured in plates containing solid YEB medium. As soon as the overlays solidified, 5 μl aliquots of the cultures (OD_600_ of 1.0) were spotted on the plates. Antagonistic effects as evidenced by reduced growth of the overlay strain around the spotted strains was evaluated daily for 5 days of incubation at 28°C.

### Modulation of Plant Development Assays

Samples from asymptomatic “UCB1” trees and plants displaying PBTS symptoms were collected for morphological analyses. Fresh petiole, stem, and root tissues were dissected into 2.5% glutaraldehyde, 0.1 M imidazole buffer (pH 7.2) and fixed overnight at room temperature. Subsequently, the fixed samples were washed with 0.1 M imidazole for 30 min, stained with 0.1 M imidazole:OsO_4_ for 2 h, washed and dehydrated to 100% ethanol, followed by a final 100% acetone step; all steps were done at room temperature. The samples were embedded into Spurrs resin and polymerized overnight at 60°C. Semi-thin 250 μm sections obtained with a UC6 ultramicrotome (Leica), were stained with toluidine blue and analyzed with a stereoscope M165FC equipped with a DFC 310 FX CCD color camera (Leica).

For the evaluation of indole-3-acetic acid (IAA) degradation, 5 mM IAA was tested as carbon source (Scott et al., 2013; Belimov et al., 2014). PBTS1, PBTS2 and D188 cultures, grown at 28°C in YEB for 2 days, were centrifuged for 2 min at 14,000 rpm, washed twice, and suspended in sterile water. These suspensions were used to inoculate MinA medium (Miller, 1972) to reach a start OD_600_ of 0.01. Growth was measured after 2 and 4 days and compared to similar cultures grown in MinA medium without a carbon source or with sucrose as controls.

Auxin production was tested with a colorimetric plate assay using Salkowski reagent. PBTS1, PBTS2, and D188 cells of 2 days old YEB cultures were washed with water and resuspended in JM medium (Crespi et al., 1992) (OD_600_ of 0.1) supplemented with 500 μg/ml tryptophan or 10 μg/ml tryptophol (indole-3-ethanol) and incubated at 28°C for 2 days. Then, 100 μl of the cultures were mixed with 100 μl Salkowski reagent R1 consisting of 12 g/l FeCl_3_ in 7.9 M H_2_SO_4_. After 30 min the pink coloration of the supernatant was measured at 540 nm (Glickman and Dessaux, 1995), neither tryptophan nor tryptophol reacted with the reagent. The production of indolic compounds was normalized to the OD_600_ of the respective cultures and the auxin concentration was calculated using a standard curve based on an IAA concentration range between 0 and 25 μg evaluated with the same protocol. The experiments were done three times.

For the assessment of interference with ethylene accumulation, 1-amino-cyclopropane-1-carboxylic acid (ACC) was utilized as a nitrogen source as described (Francis et al., 2016).

Ethylene production by PBTS1, PBTS2, and D188 was evaluated with the *Arabidopsis* triple response assay in split plates (Guzmán and Ecker, 1990). Half of the plates were filled with 10 ml PDA, and the other half was filled with 10 ml half-strength Murashige and Skoog (½MS) medium, supplemented with 0.5 g/l MES monohydrate, 0.1 g/l myo-inositol, 20 g/l sucrose, and 7.0 g/l Phyto agar (P1003; Duchefa, Haarlem, The Netherlands) (pH 5.7). The bacterial strains were inoculated on the PDA, whereas 10 *Arabidopsis* Col-0 seeds, stratified in the dark for 4 days at 4°C, were transferred to the MS medium. The center partition of the bipartite I-plates (90 × 14.2 mm; Plastiques Gosselin, Borre, France) ensured a physical separation of the bacterial cultures and the seeds, allowing only gaseous exchange via the headspace. In the positive control plates, the ethylene-releasing agent ethephon (Agrichim, Pescara, Italy) was applied either in the MS medium or applied on top of the PDA medium at a final concentration of 60 μM. A control treatment consisting of blank PDA was included as well. Plates were sealed with air-permeable plastic foil, exposed to 45 μmol/m^2^/s light for a period of 2 h at 22°C to synchronize seed germination, and subsequently grown in darkness for the remaining time of the experiment. After 7 days, seedlings were evaluated with respect to hypocotyl length and apical hook formation. The assay was conducted once with one biological replicate of 10 plants each.

The effect of emitted volatiles on plant development was tested in split plates in which two compartments were filled with 10 ml PDA and the other two with the same volume of ½MS medium without sucrose. PBTS1, PBTS2, or D188 bacteria were streak-inoculated from available master plates and allowed to grow for 2 days at 28°C. Then, 2 weeks old *N. tabacum* W38 seedlings and 1 week old *Arabidopsis* Col-0 seedlings, axenically grown on ½MS with 1% sucrose, were transferred to the ½MS compartments (6 and 5 seedlings, respectively). The plates were sealed with air-permeable plastic foil and placed in a growth chamber at 22°C under a 16 h light/8 h dark photoperiod (45 μmol/m^2^/s light irradiance from cool-white fluorescent tungsten tubes). The plants were evaluated after 11 days of growth: pictures were taken, the shoots were excised and their fresh weight measured, where after a second picture was taken to document the root system.

## Results and Discussion

### General Features and Comparative Genomics of the PBTS1 and PBTS2 Genomic Sequences

Previously, we have published the assembled draft genome sequences of PBTS1 and PBTS2 and the full genome of D188 obtained with the PacBio technology (Stamler et al., 2016). Here we analyzed the structural and functional features of these sequences (Table 1). PBTS1 has the smallest circular chromosome of the three strains consisting of 4,251,687 bp with 3,917 predicted protein-coding genes and the highest GC content of 70%; no plasmid sequences were detected. The circular chromosomes of PBTS2 and D188 have similar characteristics, 5,179,353 bp and 5,139,988 bp in size, with 4,784 and 4,747 predicted protein coding sequences, respectively, and a GC content of 64.7%. Members of the genus *Rhodococcus* have three to six copies of the ribosomal RNA (*rrn*) operon, with an average of four^7^. The number of *rrn* operons of the three strains is in line with these findings. The strains have a comparable number of transfer (t)RNAs, suggesting an equal ability to respond to favorable conditions and a similar efficiency to utilize resources (Lee et al., 2009).

**TABLE 1:** General features of the genomic sequences of strains PBTS1, PBTS2, and D188.

Feature	Chromosome	pD188-like plasmid	pFiD188-like plasmid	References
**PBTS1^a^**				
Replicon size (bp)	4251687	Absent	Absent/lost	Stamler et al., 2016
GC content	70.0%			
Protein coding genes	3917			
(CDS)				
Genes assigned to COG	2817 (71.9%)			
ncRNAs	3			
CRISPR loci	5			
rRNAs	9			
5S-16S-23S	3			
tRNAs	45			

**PBTS2^b^**				
Replicon size (bp)	5179353	137595	Absent/lost	Stamler et al., 2016
GC content	64.7%	64.7%		
Protein coding genes	4784	140		
(CDS)				
Genes assigned to COG	3605 (75.4%)	49 (35.0%)		
ncRNAs	3	0		
CRISPR loci	3	0		
rRNAs	12	0		
5S-16S-23S	4	0		
tRNAs	46	0		

**D188^c^**				
Replicon size (bp)	5139988	164725	198917	Francis et al., 2012; Stamler et al., 2016
GC content	64.7%	64.3%	61.8%	
Protein coding genes	4747	173	184	
(CDS)				
Genes assigned to COG	3509 (73.9%)	69 (39.9%)	70 (38.0%)	
ncRNAs	3	0	0	
CRISPR loci	2	0	0	
rRNAs	12	0	0	
5S-16S-23S	4	0	0	
tRNAs	46	0	0	

**^*a*^NCBI accession chromosome: NZ_CP015219. ^*b*^NCBI accession chromosome: NZ_CP015220; pD188-like plasmid: NZ_CP015221. ^*c*^NCBI accession chromosome: NZ_CP015235; pD188: NZ_CP015237; pFiD188: JN093097 and NZ_CP015236.**

Classification of the protein-coding genes into clusters of orthologous groups (COG) assigned 71.9% of the PBTS1, 75.4% of the PBTS2, and 73.9% of the D188 chromosomal sequences into functional classes. Overall, in the three strains, the COG distribution of the chromosome-coding sequences was equivalent. Regarding well-characterized classes, the categories of amino acid (E), carbohydrate (G), and inorganic (P) transport and metabolism, and the transcription category (K), had the highest representation (Table 2).

**TABLE 2:** Functional cluster of orthologous genes (COG) classification of predicted protein coding genes on the different replicons of strains PBTS1, PBTS2, and D188.

		PBTS1	PBTS2		D188		
COG functional class		Chromosome	Chromosome	pD188-like plasmid	Chromosome	pD188	pFiD188
**Metabolism**							
C	Energy production and conversion	297(7.6%)	416(8.7%)	5(3.6%)	394(8.3%)	6(3.5%)	7(3.8%)
E	Amino acid transport and metabolism	472(12.1%)	676(14.4%)	1(0.7%)	644(13.6%)	6(3.5%)	11(6.0%)
F	Nucleotide transport and metabolism	94(2.4%)	127(2.7%)	0	126(2.7%)	0	2(1.1%)
G	Carbohydrate transport and metabolism	354(9.0%)	457(9.6%)	0	440(9.3%)	3(1.7%)	5(2.7%)
H	Coenzyme transport and metabolism	255(6.5%)	324(6.8%)	0	320(6.7%)	2(1.2%)	5(2.7%)
I	Lipid transport and metabolism	235(6.0%)	310(6.5%)	0	295(6.2%)	2(1.2%)	8(4.3%)
P	Inorganic transport and metabolism	339(8.7%)	456(9.5%)	8(5.7%)	443(9.3%)	12(6.9%)	1(0.5%)
Q	Secondary metabolites biosynthesis, transport and catabolism	257(6.6%)	340(7.1%)	1(0.7%)	320(6.7%)	5(2.9%)	11(6.0%)

**Cellular processes and signaling**							
D	Cell cycle control, cell division, chromosome partitioning	79(2.0%)	94(2.0%)	4(2.9%)	95(2.0%)	5(2.9%)	7(3.8%)
M	Cell wall/membrane/envelope biogenesis	202(5.2%)	221(4.6%)	5(3.6%)	220(4.6%)	9(5.2%)	4(2.2%)
N	Cell motility	22(0.6%)	26(0.5%)	0	24(0.5%)	0	2(1.1%)
O	Post-translational modification, protein turnover, chaperones	142(3.6%)	182(3.8%)	10(7.1%)	178(3.7%)	11(6.4%)	0
T	Signal transduction mechanisms	174(4.4%)	225(4.7%)	0	221(4.7%)	1(0.6%)	5(2.7%)
U	Intracellular trafficking, secretion and vesicular transport	34(0.9%)	39(0.8%)	1(0.7%)	38(0.8%)	3(1.7%)	2(1.1%)
V	Defense mechanisms	115(2.9%)	150(3.1%)	1(0.7%)	146(3.1%)	1(0.6%)	0
W	Extracellular structures	0	0	0	0	0	0

**Information storage and processing**							
A	RNA processing and modification	1(0.03%)	1(0.2%)	0	1(0.02%)	0	0
B	Chromatin structure and dynamics	1(0.03%)	1(0.2%)	0	1(0.02%)	0	0
J	Translation, ribosomal structure and biogenesis	187(4.8%)	201(4.2%)	0	203(4.3%)	0	3(1.6%)
K	Transcription	285(7.3%)	449(9.4%)	6(4.3%)	444(9.4%)	7(4.0%)	9(4.9%)
L	Replication, recombination and repair	181(4.6%)	162(3.4%)	6(4.3%)	172(3.6%)	9(5.2%)	4(2.2%)

**Poorly characterized**							
R	General function prediction only	699(17.8%)	929(19.4%)	9(6.4%)	900(19.0%)	12(6.9%)	13(7.1%)
S	Function unknown	218(5.6%)	286(6.0%)	9(6.4%)	281(6.0%)	7(4.0%)	3(1.6%)

**The number of coding sequences in each functional class is given; between brackets is the relative percentage of each category in relation to the total number of coding sequences.**

**FIGURE 1:**
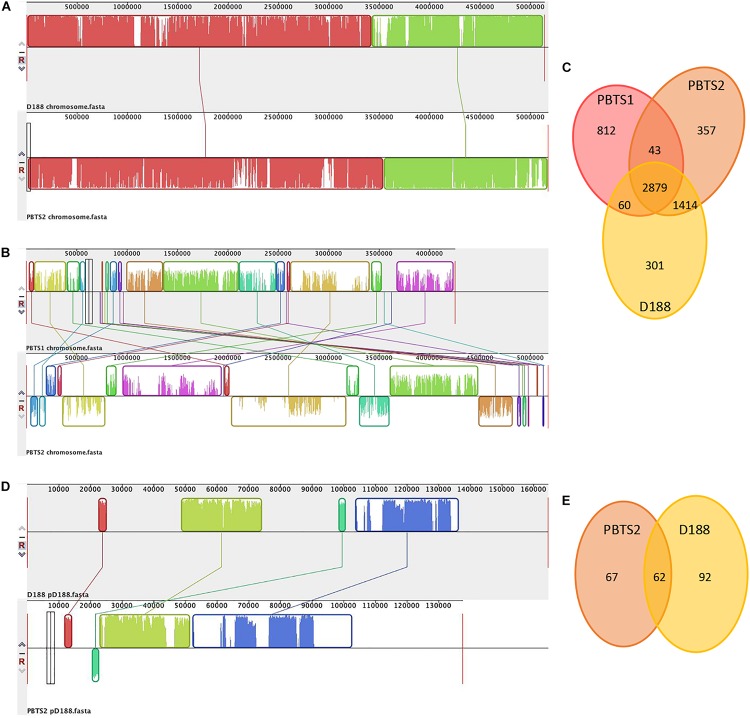
Comparison of the chromosomes of PBTS1, PBTS2, and D188, and the pD188-like plasmids of PBTS2 and D188. **(A)** Mauve alignments of the chromosomes of PBTS2 and D188, **(B)** those of both PBTS isolates, and **(D)** the two pD188-like plasmids analyzed in this study. **(C)** Venn diagrams of orthologous groups (as derived by Reciprocal Smallest Distance) indicating the shared and unique genes among the three *Rhodococcus* chromosomes and **(E)** the two pD188-like plasmids.

Alignment of the chromosome sequences showed that the PBTS2 and D188 chromosomes were highly co-linear (Figure 1A). Although PBTS2 also shared a significant amount of genetic characteristics with PBTS1, the syntenic regions in this case were smaller and more scattered due to inversions and translocations (Figure 1B). Based on the chromosome sequences, the core genome was calculated with OrthoMCL v. 1.4 (Li et al., 2003). The three strains shared 2,879 orthologous groups representing 76% of the PBTS1 proteome, 61% of that of PBTS2, and 63% of that of D188; PBTS2 and D188 additionally shared 1,414 orthologous groups, corresponding in total to a 92-93% overlap in their proteomes (Figure 1C). Of the three strains, PBTS1 had the highest number of strain-specific coding sequences, 812 orthologous groups (870 genes; Supplementary Table S2) representing 21% of its chromosomal genes. Both *R. fascians* strains only had 6-8% of unique genes (381 for PBTS2 (Supplementary Table S3); 320 for D188). Considering putative differences in host range, namely woody versus herbaceous for PBTS and D188, respectively, we were particularly interested in the 43 orthologous groups that were conserved in both PBTS isolates, but lacking in D188. However, amongst the unique PBTS genes, none encoded obvious host range-related functions (Supplementary Table S4).

Besides the circular chromosome, PBTS2 and D188 have a circular plasmid that has been designated pD188 and is not associated with pathogenicity in the latter (Desomer et al., 1988). In D188, pD188 is 164,725 bp in size with a 64.3% GC content and putatively encodes 173 proteins, of which 39.9% were assigned to a COG category (Table 1). The pD188-like plasmid in PBTS2 has 137,595 bp with a 64.7% GC content and 140 protein-encoding genes, of which 35% were classified in a COG category (Table 1). Although the COG distribution in both plasmids is quite different, in both cases the most represented classes are involved in post-translational modification, protein turnover, chaperones (O), and inorganic transport and metabolism (P) (Table 2). Alignment of both plasmid sequences and calculation of their shared coding sequences showed a limited co-linearity (Figure 1D). Only 62 orthologous groups were conserved (Figure 1E), among which genes that confer resistance to copper and cadmium (Supplementary Table S5).

Finally, D188 possesses additionally the virulence-associated linear plasmid pFiD188 (Francis et al., 2012) that is 198,917 bp in size with a 61.8% GC content and 184 protein-encoding genes (Table 1). COG annotation classified 38% of the coding sequences with the classes involved in amino acid transport and metabolism (E), and secondary metabolites biosynthesis, transport and catabolism (Q) most strongly represented (Table 2).

### The Pathogenicity of PBTS1 and PBTS2 Is Linked With the Presence of *fas* Sequences

**FIGURE 2:**
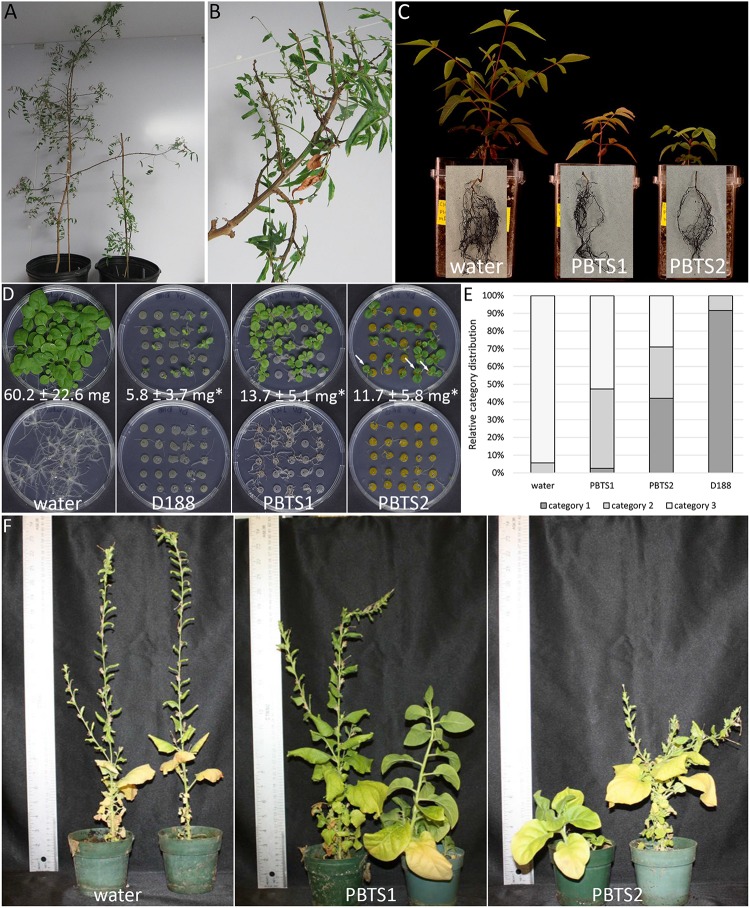
Reassessment of the pathogenicity of PBTS1 and PBTS2 on different hosts. **(A)** Control (left) and PBTS shoot-inoculated (right) “UCB-1” clonal plants. Note the strongly stunted growth after shoot inoculation with a mixture of PBTS1 and PBTS2 (12 months after infection). **(B)** Detail of co-infected shoot showing loss of apical dominance and formation of deformed shoots and leaves. **(C)** Stunted shoot growth and reduced root system of PBTS1 and PBTS2 infected “UCB-1” seedlings (45 days after infection; *n* = 3). At the end of the experiment, the plants were removed from the pots and the substrate was washed from the roots; root pictures are superimposed on those of the respective plants. **(D)** Seedling assay to monitor the effect of PBTS1, PBTS2, and D188 on the development of *N. tabacum* seedling shoots (top) and roots (bottom). The arrows in the PBTS2 panel indicate seedlings with leaf deformations. The average shoot fresh weight (FW) as measured at the end of the experiment is indicated (14 days after infection). Statistical differences between infected plants and the mock-infected control were calculated using Student’s *t*-tests (*P < 0.01; *n* = 14–22). **(E)** Quantification of the growth inhibition effect shown in **(D)**. Category 1, full growth inhibition with arrest at the cotyledon stage; Category 2, intermediate growth inhibition; Category 3, no growth inhibition. **(F)** Severe stunting of *N. benthamiana* plants grown in substrate inoculated with PBTS1 or PBTS2 (3 months after infection; *n* = 5).

The pathogenicity of PBTS1 and PBTS2 was re-evaluated on “UCB-1” clonal plants and seedlings and was additionally tested on two tobacco species. “UCB-1” clonal plants were co-inoculated with PBTS1 and PBTS2 cultures, placed in a plant incubator, and their development followed over a period of 12 months. In agreement with our previous findings (Stamler et al., 2015b), control plants grew as expected (Figure 2A), whereas PBTS-infected trees showed severe stunting (Figure 2A) and a loss of apical dominance, resulting in the outgrowth of deformed short branches with small distorted leaves (Figure 2B). Unexpectedly, in an independent experiment, none of the 25 clonal “UCB-1” plants dip-infected with a mixture of PBTS1 and PBTS2 developed symptoms, although the plants exhibited vigorous growth (Supplementary Figure S1). Subsequent qPCR analysis demonstrated that the PBTS1 culture did not contain any of the tested *fas* genes. In PBTS2, the Cq value for the 16S rRNA gene, used as a reference, was approximately 14, whereas very high Cq values ranging between 36 and 39 were obtained for *fasC*, *fasE*, and *fasF* signifying that few cells in the culture had these genes. Similarly, whereas inoculation of “UCB-1” seedlings with either of the PBTS isolates resulted in stunted growth of the shoots and reduced development of the root system in one experiment (Figure 2C), no symptoms developed on any of the inoculated plants in a subsequent experiment with the same setup (data not shown); the presence of the *fas* genes was not evaluated in these experiments.

As a first assessment of putative host range restrictions, the PBTS strains were used to infect two tobacco species, which are known to be highly responsive to leafy gall-inducing *R. fascians* isolates. In seedling infection assays with *Nicotiana tabacum*, plant responses have been categorized in 3 classes that determine the pathogenicity level of the strain under study (Maes et al., 2001). Infection with D188, used as reference, completely blocked seedling development at the cotyledon stage and fully inhibited root formation (Figure 2D), consistent with previous findings (Crespi et al., 1992; Temmerman et al., 2000; Vereecke et al., 2000, 2002); nearly all plants fell in category 1 (Figure 2E). Interestingly, both PBTS isolates also caused developmental effects in the tobacco host, but, in contrast to D188, the results were more variable. Inoculation with PBTS1 resulted in an almost equal distribution of plants exhibiting an intermediate (category 2) or no (category 3) growth inhibition, with less than 3% of the plants represented in category 1 (Figure 2E). Besides their growth retardation, none of the shoots exhibited abnormal morphologies, but the root systems were significantly reduced when compared to non-infected controls (Figure 2D). Upon infection with PBTS2, over 40% of the plants fell in category 1 and 28% in categories 2 and 3 (Figure 2E). Interestingly, PBTS2 infection also resulted in leaf deformations in up to 60% of the plants and inhibited root formation almost to the same extent as D188 (Figure 2D). Determination of the fresh weight of the plants revealed that D188 decreased the biomass of the plants to 10% of that of the non-infected controls, whereas plants infected with either PBTS1 or PBTS2 reached a fresh weight that was approximately 20% of that of the uninfected controls (Figure 2E). In line with these findings, when seedlings of *N. benthamiana*, which has also been used as a host for leafy gall inducers (Creason et al., 2014), were planted in substrate co-inoculated with PBTS1 and PBTS2, their growth was severely stunted, flowering was delayed, and the number of flowers was reduced (Figure 2F).

Altogether these results confirm the pathogenic identity of PBTS1 and PBTS2 and extend their host range past pistachio. However, not all infections consistently provoked symptom development and the maintenance of *fas* genes in the inoculum was seemingly an issue that might be at the basis of the observed variability. This limited occurrence of *fas* genes in the PBTS cultures concurs with the absence of *fasA* and *fasD* in the genomic sequences of both PBTS1 and PBTS2 and the absence of the *vicA* homolog identified by PCR in the PBTS1 sequence (Stamler et al., 2015b). From what follows, inoculum preparation appears to have an important impact on the presence or absence of these putative virulence genes in the PBTS cultures and thus on the outcome of the infections.

### Evidence for the Presence and Loss of pFi-Related Sequences in PBTS1 and PBTS2 Cultures

From the above and in contrast to the leafy gall-inducing model strain D188, maintenance of the *fas* genes does not seem to be a stable characteristic in the PBTS isolates. To get insight into this matter, we examined by qPCR the number of cells within pure cultures of PBTS1, PBTS2, and D188 that contain the chromosomal *vicA* gene (*vicA2* in PBTS1) and the *fasD* gene that is located on the conjugative linear plasmid pFiD188 in strain D188 (Crespi et al., 1992). PBTS1 has two homologs of the malate synthase gene: *vicA2* which is the chromosomal counterpart of *vicA* in PBTS2 and D188, and *vicA* which should be considered a pathogenicity gene in this isolate (Stamler et al., 2015b; Savory et al., 2017). The results indicated that the number of cells that contained *vicA* was comparable for the three strains (*vicA2* in PBTS1), but that a significant discrepancy occurred amongst strains for the presence of cells positive for *fasD* (and *vicA* for PBTS1). Whereas the ratio for *fasD*- and *vicA*-positive cells was 1:1 in D188 cultures, for PBTS1 only 1 in 10,000 *vicA2*-positive cells contained the *fasD* and the *vicA* gene, and for PBTS2 only 1 in 10,000 cells contained both *fasD* and *vicA*. In an independent, indicative qPCR experiment, the 16S rRNA gene was used as a reference and the presence of three genes of the *fas* operon, *fasC*, *fasE* and *fasF*, was assayed in D188 cultures and in three different isolates of each PBTS species. The number of D188 cells that carried the three *fas* genes and the 16S rRNA gene was 100%, corroborating the presence of pFiD188 in each of the cells. For the PBTS1 isolates, however, on average only 8% of the cells had *fasC* (4.4, 8.8, and 12%), 4.9% *fasE* (1.5, 5.0, and 8.3%), and 2.8% *fasF* (1.6, 2.2, and 4.7%). For the PBTS2 isolates the number of cells with the *fas* genes was even lower ranging between 1.5 and 2.3 cells in 10,000 (*fasC*: 1.6, 1.8, and 3.3 in 10,000; *fasE*: 1.7, 1.5, and 4.6 in 10,000; *fasF*: 1.1, 1.2, and 2.1 in 10,000). Sequence determination of the *vic* and *fas* amplicons of both experiments confirmed their identity (Supplementary Figure S2).

The finding that only a subset of PBTS1 and PBTS2 cells retain *fas* genes when cultured was further supported by sequencing two additional PBTS1-like (Rhodo 11 and Rhodo 13) and PBTS2-like isolates (Rhodo 10 and Rhodo 12) with the Illumina technology. Calculation of pairwise ANI and *is*DDH values, revealed that the genomic sequences of the two PBTS1-like isolates were nearly identical to the published PBTS1 sequence, but very different from the genome sequence of *R. corynebacterioides* NBRC 14404 (Supplementary Figure S3). In fact, despite the 99.73% identity of their 16S rRNA sequences, the obtained ANI and *is*DDH values were well below the respective 94% and 70% threshold values determined for species delineation (Sangal et al., 2016), indicating that PBTS1 should not be considered a *R. corynebacterioides* strain. In contrast, whereas the genomes of the two PBTS2-like isolates were identical based on the ANI and *is*DDH values, they diverged more from the published PBTS2 and the D188 sequence, but were still well within the range to be considered the same species (Supplementary Figure S3); hence, PBTS2 should be considered a *R. fascians* strain. When the reads were assembled, no pFi-like replicon was identified in any of the four isolates, concurring with the published data (Stamler et al., 2016). However, by assuming that only a fraction of the cells in the cultures used for sequencing would contain virulence genes, the putative virulence replicon would probably not be assembled. In line with this reasoning, we mapped all the reads to pFiD188. For Rhodo 12 and Rhodo 13, a single hit was obtained, corresponding to *pFi_052* and *attG*, respectively (Supplementary Table S6), and none for Rhodo 11. However, with Rhodo 10, 15 hits were obtained yielding sequences of 2,092 bp identical and 378 bp similar to pFiD188 (83–98% identical) (Supplementary Table S6). The hits were spread throughout the pFiD188 sequence and comprised three loci of the conserved R regions and eight loci of the unique U regions (Francis et al., 2012), including *fasA*, *fasF*, *nrp2*, *stk4*, and *stk5* (Supplementary Table S6). Whereas, based on these data, no hypothesis can be put forward for PBTS1, we could speculate that PBTS2 has a virulence carrier resembling pFiD188, but this hypothesis requires further experimental confirmation. We then used the reads to calculate the number of cells with such a putative replicon in the cultures used for genomic sequencing: the obtained numbers were 1:200,000 cells (15 out of 3,324,630 pairs) for Rhodo 10, 1:4,000,000 cells (1 out 3,859,253 pairs) for Rhodo 13 and 1:3,000,000 for Rhodo 12 (1 out 31677999 pairs), which is even lower than the results obtained in the qPCR experiments.

**FIGURE 3:**
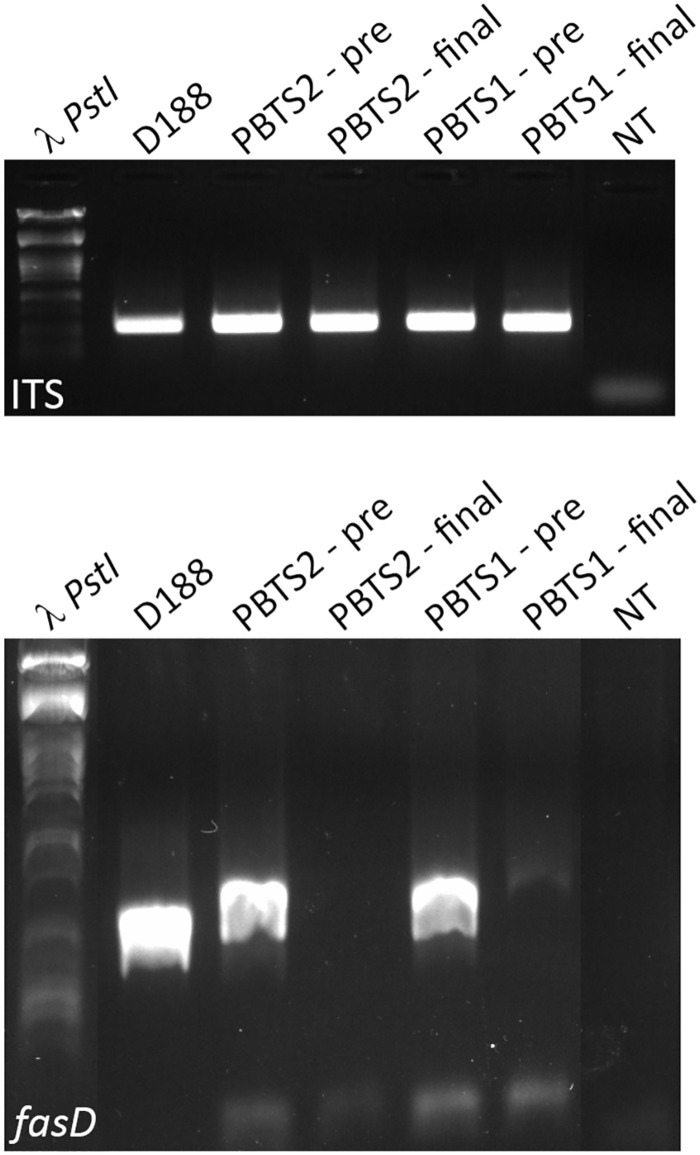
Presence and loss of *fasD* in PBTS1, PBTS2, and D188 cultures. PCR analysis with ITS amplification to validate DNA quality, equal concentration, and stability. Amplification of *fasD* shows its presence in the PBTS1 and PBTS2 pre-cultures (albeit in fewer cells than in D188), and its loss in the PBTS isolates after a single additional passage in rich liquid medium. λ PstI, DNA ladder; NT, no template control. The images of the original gels have been cut and rearranged for a more logic order of the samples. The original images have been provided to the reviewers for evaluation.

From the above, we postulated that the *fas* genes in the PBTS isolates were lost upon repeated sub-culturing in rich medium. To test this hypothesis, 5 ml YEB cultures of PBTS1 and PBTS2 were initiated from glycerol stocks. After 2 days of growth at 28°C, a 1-ml sample was taken for gDNA extraction and the remainder of the pre-culture was diluted in 500 ml YEB and grown for another 2 days, where after gDNA was extracted from a 1 ml sample. Ten ng of each gDNA was used for endpoint PCR analysis of *fasD* and the ITS sequence as a reference; gDNA from D188 was used as a control. A fragment of comparable intensity was detected for the ITS amplicon in all 5 cultures. In accordance with the previous experiments, a *fasD* amplicon was detected in PBTS1 and PBTS2 pre-cultures, but the intensity of the fragment was weaker than in D188, indicating that it is present only in a sub-fraction of the PBTS cells (Figure 3). The identity of the *fasD* amplicon was confirmed by sequencing (Supplementary Figure S2). Importantly, after a single additional passage in rich medium the presence of *fasD* could no longer be detected in both PBTS cultures (Figure 3).

**FIGURE 4:**
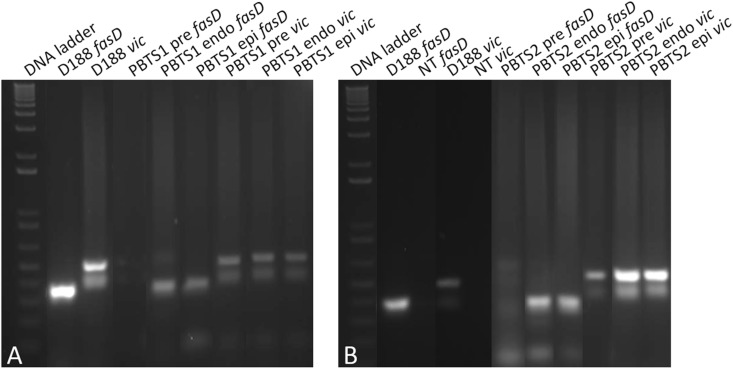
Evidence for the *in planta* enrichment of *fasD* in PBTS1 and PBTS2. **(A)** Differential amplification of *fasD* but not *vic* in PBTS1 and **(B)** PBTS2 bacteria originating from broth pre-cultures (pre) and in endophytic (endo) and epiphytic (epi) cells re-isolated from the *N. benthamiana* host; amplification in D188 is used as a reference. DNA ladder: 1 Kb Plus ladder; NT, no template control. The images of the original gels have been cut and rearranged for a more logic order of the samples. The original images have been provided to the reviewers for evaluation.

We then hypothesized that the sub-population of *fasD*-positive cells in the PBTS1 and PBTS2 cultures would increase when the bacteria were in contact with a plant host. In support of this postulation, *fasD* and *vic* could be amplified by qPCR in gDNA extracted from leaf and stem samples of field grown “UCB-1” rootstock trees exhibiting PBTS symptoms (data not shown). Next, liquid PBTS cultures were used to inoculate *N. benthamiana* plants and a sample of these start cultures was taken for gDNA extraction. Three weeks after infection epiphytic colonies were isolated from plant tissue washes and endophytic colonies from surface sterilized plant tissue; gDNA was extracted from these samples. The gDNA from the starting cultures and from the re-isolated bacteria was subjected to PCR for the amplification of *vic* and *fasD*. For both PBTS1 and PBTS2, hardly any amplification of *fasD* was detected in the starting cultures, although the *vic* gene was clearly present in the populations. However, a single passage on the host resulted in a clear enrichment of *fasD* in epi- and endophytic cells (Figures 4A,B).

Altogether these data provide strong evidence that the maintenance of the putative virulence genes in the PBTS strains is unstable in an *ex planta* environment. Loss of virulence is actually a recurrent observation for all kinds of pathogens (e.g., Krokene and Solheim, 2001; Wright et al., 2003; Soto et al., 2006; Mitra, 2015) and although the presence of the linear virulence plasmid is a very solid characteristic in many leafy gall inducers, virulence instability has been recorded for *R. fascians* as well. Already in 1948, Margaret Lacey, a “leafy gall” research pioneer, reported that anti-auxin activity in some *R. fascians* isolates was lost by cultivation on artificial media (Lacey, 1948). Jacobs and Mohanty (1951) observed that several virulent *R. fascians* strains that had retained their pathogenicity at least 5–6 years after isolation, would become non-pathogenic after longer laboratory cultivation periods. Growth of *R. fascians* isolates on rich media at 37°C resulted in a complete loss of virulence (Sabart et al., 1986). Even strain D188 could be cured from its linear plasmid by growth at 37°C followed by other harsh treatments (Desomer et al., 1988). Additionally, plasmid instability has also been observed in *R. fascians* isolates in the absence of non-permissive growth conditions. For instance, Nikolaeva et al. (2009) showed that different isolates from symptomatic ornamental plants exhibited a varying degree of pathogenicity (±35% pathogenic, ±30% non-pathogenic, and ±35% with a variable phenotype). When single colonies from individual pure isolates exhibiting variable pathogenicity were analyzed, they turned out to consist of two sub-populations, one with *fasD* and one without *fasD*; bacteria without *fasD* lacked all tested linear plasmid markers (Nikolaeva et al., 2009). It would be interesting to compare the chromosomal and plasmid sequences of some of the unstable isolates to those of D188 to identify putative stability/instability determinants, but as far as we know, their genomes have not been sequenced. Intriguingly, as opposed to the loss of virulence upon growth outside the host, both Lacey (1948) and Sabart et al. (1986) noticed that re-isolated bacteria obtained from plants inoculated with weakly active (auxin degradation) or “regular” virulent *R. fascians* isolates, exhibited a strong increase in auxin degradation potential or a hypervirulent character, respectively. These findings are in line with our observation of *in planta* enrichment of *fasD*-positive PBTS isolates.

The instability of the *fas* genes in the PBTS isolates will make the study of their pathogenicity strategies very challenging. Nevertheless, pending the identification of their virulence carrier, we explored their chromosome sequences for genes that might be implicated in the adaptation to their habitats and their apparent co-occurrence or that may play a role in the interaction with their host. Most of these features were functionally assessed to validate the *in silico* predictions.

### The Metabolic Features of PBTS1 and PBTS2 Suggest That Nutritional Resource Partitioning Probably Contributes to Their Apparent Co-occurrence

More than 20% of the coding sequences in the two PBTS isolates belong to the COG annotation class T “Signal transduction mechanisms” (Table 1) and the repertoire of two-component systems consists of 18 gene pairs (histidine kinase/response regulator) in PBTS1 and 23 in PBTS2 (Supplementary Table S8), suggesting that both bacteria probably have a strong ability to respond to changes in their surrounding and can thrive in diverse environments. Additionally, both bacteria have an extensive set of ABC- (Supplementary Table S8) and MSF-type transporters (Supplementary Table S9) indicating that they might benefit from the availability of various potential N and C sources in their habitat, including mannitol, ribose, glucose, betaine, choline, and diverse amino acids that are all released by plants as root exudates (Faure et al., 2009). KEGG analysis of the chromosome sequences of PBTS1 and PBTS2 also predicted the presence of several central metabolism pathways, including glycolysis, pyruvate metabolism, the Entner-Doudoroff pathway, the Krebs cycle, and the pentose phosphate pathway, implying that the PBTS bacteria are capable of using the wide variety of carbon sources internalized by the transporters. Interestingly, based on these analyses, PBTS1 and PBTS2 would be unable to utilize galactose and methionine and inositol, xylose, and citrate, respectively. Such a differential utilization of nutrient sources that are putatively available in the habitat might represent one mechanism to support the co-occurrence of the PBTS strains (Schlechter et al., 2019).

**FIGURE 5:**
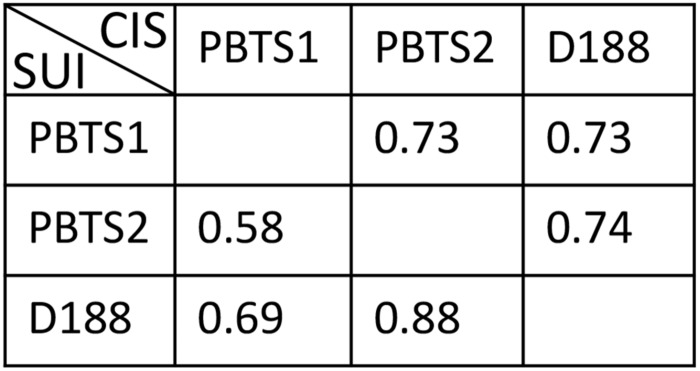
Calculated niche overlap indices based on the utilization of diverse carbohydrates, amino acids, and hormones as carbon sources and ACC as nitrogen source. SUI, strain under investigation; CIS, co-inhabiting strain.

This prediction was tested by evaluating the nutrient utilization patterns of PBTS1 and PBTS2. To this end, the PBTS bacteria were grown in minimal medium supplemented with a 0.5% final concentration of either one of 22 different carbohydrates or either one of 12 amino acids as carbon sources; strain D188 was included as reference. In agreement with the sequence data, PBTS1 was unable to grow on methionine or galactose and PBTS2 did not proliferate on myo-inositol. However, PBTS2 did grow on citrate (Table 3). Since there were quite some differences in the carbon source utilization profiles of the three strains (Table 3), we calculated niche overlap indices (NOIs) to estimate niche similarity of the PBTS strains and D188. As described by Wilson and Lindow (1994), for each pair of strains NOIs were calculated as the number of carbon sources utilized by both strains as a proportion of the total number of carbon sources utilized by the strain in question. The smallest niche overlap was between PBTS2 and PBTS1 (Figure 5), suggesting that the nutritional competition between both strains might not interfere with their co-habitation of the same niche.

**TABLE 3:** Utilization patterns of a selection of carbon sources for PBTS1, PBTS2, and D188.

Carbon source	PBTS1	PBTS2	D188
**References**			
None	**−**	**−**	**−**
YEB	**++++**	**++++**	**++++**
(NH_4_)_2_SO_4_*	++++	++++	++++

**Carbohydrates**			
Arabinose	**−**	**+++**	++
Cellobiose	**−**	**−**	**−**
Citrate	**−**	++	**±**
CMC	**−**	**−**	**−**
Fructose	+++	++++	++
Galactose	**−**	**+++**	**±**
Glucose	**+**	**±**	**++**
Glycerol	**+++**	++++	**±**
Lactose	**−**	**−**	**++**
Malt extract	**+++**	**++++**	**++++**
Maltose	**−**	**−**	**++**
Mannitol	**++++**	**++++**	+++
Mannose	+	++	**±**
Myo-inositol	++++	**−**	**−**
Raffmose	**−**	**−**	±
Rhamnose	±	+	**±**
Soluble starch	±	±	+
Sorbose	**−**	**−**	**−**
Sucrose	**+++**	**++++**	**+++**
Trehalose	**±**	**++**	**++**
Xylan	**±**	±	±
Xylose	+	±	±

**Amino acids**			
2-aminobutyric acid	**−**	**−**	**±**
Asparagine	±	++	+
Cysteine	**−**	**−**	**−**
Glutamine	**+**	**−**	**±**
Glycine	**+**	**−**	**−**
Histidine	**++**	+++	+
Leucine	**+++**	+++	±
Lysine	**++**	+++	+
Methionine	**−**	**±**	**±**
Proline	**+**	**+**	**+**
Serine	**−**	**−**	**−**
Tryptophan	**+**	+++	**+**

**Hormones**						
IAA	**−**	**+**	**−**
ACC*	**−**	**++**	**+**

*The data are the average of two replicates measured two days after inoculation; bold characters indicate that the bacteria had not reached stationary phase at that time point and continued to grow until 4 days after inoculation when the experiment was ended. Growth is relative to the negative control without addition of any carbon source (*−) and growth in rich YEB medium (++++). *ACC was given as nitrogen source, with sucrose as carbon source; growth on (NH_4_)_2_SO_4_ was used as a reference.

Altogether, these data indicate that the metabolic features of PBTS1 and PBTS2 would support their co-occurrence, in agreement with the consistent co-isolation of both PBTS strains from “UCB-1” trees exhibiting PBTS symptoms (Stamler et al., 2015a, b).

### Complementary Activities in PBTS2 Might Support Habitat Fitness of PBTS1

**FIGURE 6:**
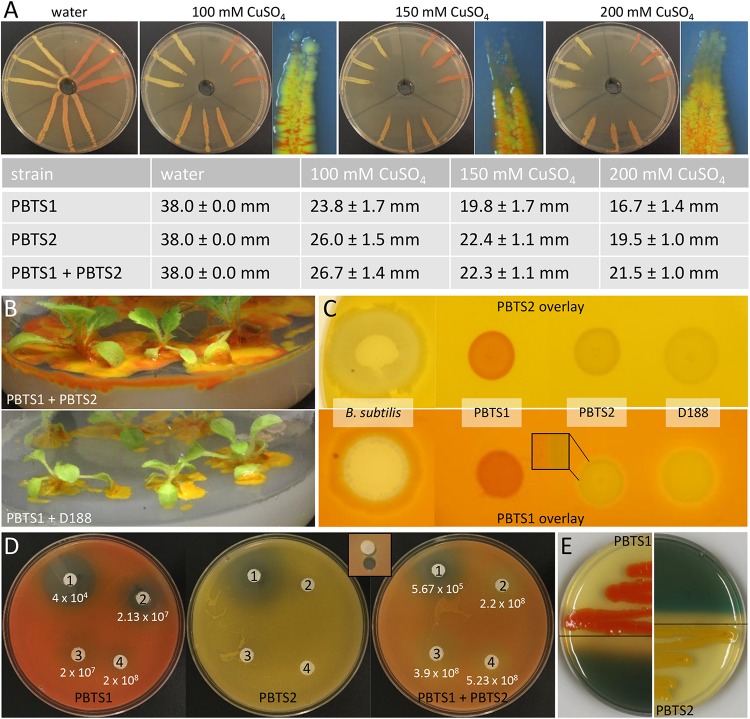
Functional evidence for competitiveness features of PBTS1 and PBTS2. **(A)** Copper resistance assayed with the plate diffusion method. The top panels represent an overview of the bacterial growth on the different CuSO_4_ concentrations and a detailed view of the tip of the growth streak near the copper source (note the presence of the orange-colored PBTS1 colonies). The table represents the average length of the growth streaks. At each copper concentration, the growth of PBTS1 in the PBTS1 and PBTS1+PBTS2 inoculation streaks was statistically different as calculated by Student’s *t*-tests (*P* < 0.01; *n* = 6). **(B,C)** Antagonistic effect of D188, but not of PBTS2, against PBTS1. **(B)** Differential growth of PBTS1 on ½MS medium after co-inoculation of *N. tabacum* with PBTS2 (top) or D188 (bottom). **(C)** Lack of antagonistic activity by any of the strains when an overlay of PBTS2 is applied (top). In case of an overlay with PBTS1, an inhibition zone is formed around D188, but not around PBTS2 (bottom); *Bacillus subtilis* was used as positive control. **(D)** Antibiograms with four penicillins (1, mezlocillin; 2, oxacillin; 3, amoxicillin; and 4, ampicillin) and overlays of PBTS1, PBTS2 or both PBTS strains together. After 3 days of growth, the PBTS1 titers on an agar plug adjacent to the antibiotic discs were determined (see inset for sampling zone). The average PBTS1 titer is indicated and was statistically different between PBTS1 and PBTS1+PBTS2 for each antibiotic (calculated using Student’s *t*-tests; *P* < 0.01; *n* = 6). **(E)** Siderophore production by PBTS1 and PBTS2 on CAS/YEB plates.

Besides niche partitioning, complementary activities involved in habitat competitiveness might also contribute to the co-occurrence of both PBTS species. Therefore, the PBTS1 and PBTS2 chromosomes were mined for the presence of genes putatively involved in bactericide resistance and antimicrobial production. To mitigate copper micronutrient deficiencies in pistachio orchards, trees are fertigated or sprayed with copper sulfate (Kallsen et al., 2000; Soliemanzadeh et al., 2014). Additionally, copper fungicides are used for the management of *Septoria* leaf spot (Michailides, 2005). As a consequence of these management practices, epiphytic PBTS populations could be subjected to toxic levels of copper. The chromosome of PBTS2 contains nine genes putatively involved in copper detoxification and active copper efflux, including two copper oxidases, a copper translocating P-type ATPase, a CopC-like chaperone, a CopD-like copper resistance protein, two copper binding proteins, a copper transporter, and a CopY-like repressor. Even more, PBTS2 has an additional copper resistance system on the pD188-like circular plasmid that consists of a CopG-like copper translocating P-type ATPase, a CopC-like chaperone, a CopD-like copper resistance protein, and a CopY-like repressor (Supplementary Table S10). Although PBTS1 also contains six genes encoding proteins related to copper resistance, including three copper chaperones, one copper translocating P-type ATPase, a CopD-like copper resistance protein, and a copper transporter, no copper oxidase was identified suggesting that efflux is the main copper homeostasis mechanism in this strain (Supplementary Table S10). Copper resistance assayed with the plate diffusion method (Hassen et al., 1998), revealed that PBTS1 was indeed more sensitive to copper than PBTS2 as the extent of the growth in the inoculated streaks was consistently significantly less for PBTS1 than for PBTS2 (Figure 6A). However, by inoculating PBTS1 together with PBTS2, PBTS1 colonies occurred at the streak tips at all copper concentrations tested, indicating that the presence of PBTS2 increases the PBTS1 tolerance to higher Cu levels (Figure 6A). Furthermore, PBTS2 possesses all the genes required for 4-hydroxybenzoate degradation, a natural antimicrobial phenolic compound produced by many plants and highly abundant in ripe pistachio hulls (Barreca et al., 2016). The pathway is organized in a more complicated super-operonic gene cluster (*pobAB*, *pcaJI*, *pcaHGBDR*, *pcaC*, and *pcaF*) that differs from that described in *Xanthomonas campestris* pv. *campestris* (Wang et al., 2015), but more importantly, in PBTS1 this detoxification mechanism is seemingly incomplete (Supplementary Table S10).

AntiSMASH analysis (Blin et al., 2019) suggested that the spectrum of bioactive compounds produced by the secondary metabolite clusters of both PBTS isolates is probably quite different (Supplementary Table S11), implying putative complementarity in their responses toward surrounding microbes. Following an experiment in which 3 weeks old axenically grown *N. tabacum* plants were dip-infected with a mixture of PBTS1 and PBTS2, the excessive growth of both bacteria observed on the plant medium clearly indicated that they did not exhibit any antagonistic effect against each other (Figure 6B). Nevertheless, when PBTS1 was co-inoculated with D188, hardly any orange-colored bacteria could be detected, suggesting that D188 produced a bioactive compound that is not synthesized by the highly similar PBTS2 (Figure 6B). Accordingly, no antagonistic activity of PBTS2 could be demonstrated against PBTS1 nor against D188 with overlay assays. When PBTS1 was used as overlay, however, the cells grew less densely around the D188 spot, but no halo developed around PBTS2 (Figure 6C). Comparison of the antiSMASH results on the PBTS2 and D188 chromosomes revealed an almost complete overlap in secondary metabolite clusters (Supplementary Table S11), with two exceptions. Cluster 12 in D188 contains a single putative type I PKS-encoding gene with an upstream gene encoding a hypothetical protein and a downstream gene encoding an aminotransferase class I/II-fold pyridoxal phosphate-dependent enzyme (*A3L23_RS14450*, *A3L23_RS14445*, and *A3L23_RS 14440*); these three genes are absent in the PBTS2 chromosome. Additionally, although cluster 3 in PBTS2 and cluster 9 in D188 are similar and putatively code for the production of a bacteriocin, PBTS2 has two extra genes and a small hypothetical gene that differs from that in D188, possibly affecting functionality (Supplementary Table S11). Further experimentation is required to determine whether these gene clusters are responsible for the observed antagonistic effect of D188 against PBTS1.

Soil dwelling microbes typically use antibiotic production, especially ß-lactams, to protect their niche in this nutrient-poor environment. Therefore, successful soil inhabitants often exhibit antibiotic resistances (Butterworth et al., 1979; Bucher et al., 2019). Both PBTS strains appeared to be characterized by a differential set of ß-lactamases (Supplementary Table S10) and, in agreement with the higher number of putative ß-lactamase genes in PBTS2 compared to PBTS1 (5 versus 2), antibiograms showed that the former was indeed much more resistant to different penicillins (Table 4). Quantification by serial dilutions of PBTS1 titers just aside antibiotic discs in antibiograms with four penicillins in overlays with either PBTS1 alone or both PBTS strains together, showed that the presence of PBTS2 enhanced the tolerance of PBTS1 for increased penicillin levels, with PBTS1 titers that were 10-fold higher for oxacillin, 14-fold for mezlocillin, and 20-fold for amoxycillin (Figure 6D). Taken together these data indicate that by associating with PBTS2, PBTS1 would strongly improve its ability to survive in soil and on pistachio plants.

**TABLE 4:** Penicillin resistance profile of PBTS1 and PBTS2.

		PBTS1		PBTS2	
Antibiotic	Concentration (^g)	Halo size^*a*^(mm ± SD)	R/S^*b*^	Halo size^*a*^(mm ± SD)	R/S^*b*^
Amoxicillin	25	23.2 ± 1.0	SS	8.8 ± 0.3	S
Ampicillin	25	20.6 ± 0.3	SS	8.2 ± 0.2	S
Azlocillin	75	27.3 ± 0.4	SS	17.6 ± 1.0	SS
Carbenicillin	100	32.4 ± 0.9	SS	15.3 ± 0.1	SS
Cloxacillin	5	6.00 ± 0.0	**RR**	6.00 ± 0.0	**RR**
Methicillin	10	6.00 ± 0.0	**RR**	6.00 ± 0.0	**RR**
Mezlocillin	75	22.5 ± 2.5	SS	21.0 ± 1.0	SS
Oxacillin	5	6.00 ± 0.0	**RR**	6.00 ± 0.0	**RR**
Penicillin	10	15.3 ± 0.6	SS	8.2 ± 0.5	S
Piperacillin	100	22.9 ± 0.4	SS	16.2 ± 0.5	SS
Ticarcillin	75	31.9 ± 3.0	SS	12.6 ± 1.7	S

**^*a*^Data are the average of two independent experiments.***^*b*^***Antibiotic resistance or sensitivity based on the halo size; RR, no halo = disc size of 6.0 mm; R, 6.1 – 7.0 mm; RS, 7.1 – 9.0 mm; S, 9.1 – 15.0 mm; SS, ≥ 15.1 mm.**

### The PBTS1 and PBTS2 Chromosomes Encode Different Iron-Capturing Mechanisms

Plant surfaces as well as soil are environments that are highly challenging and successful survival requires adaptive skills to acquire essential nutrients from the surroundings. As iron is a growth-limiting factor, plant pathogens generally have diverse mechanisms to get iron from either their host or their habitat (Lemanceau et al., 2009). As for other bacteria (Sheldon and Heinrichs, 2015), the presence of two iron-dependent repressors indicates that the iron regulon in both PBTS strains is under tight transcriptional control (Supplementary Table S10). Although the bioavailability of inorganic iron is generally low, the PBTS isolates have an inorganic iron transport system that allows them to access ferrous iron under permissive environmental conditions (Supplementary Table S10; Sheldon and Heinrichs, 2015).

AntiSMASH analysis of the chromosomes of PBTS1 and PBTS2 identified 13 and 19 regions possibly involved in secondary metabolism, respectively (Supplementary Table S11). In PBTS2, cluster 9 was highly similar to the siderophore operon previously identified in the D188 chromosome (Francis et al., 2016). This operon consists of 17 genes encompassing three non-ribosomal peptide synthases (NRPSs), a salicylate synthase, three siderophore-interacting proteins, and two composite ABC transporters (Supplementary Table S10) that are related to enzymes involved in mycobactin production, a mycobacterial salicylic acid-based siderophore essential for pathogenicity (De Voss et al., 2000; Reddy et al., 2013). In agreement with this finding, siderophore activity could indeed be demonstrated on Chrome Azurol S (CAS)/YEB plates (Figure 6E).

Regarding siderophore production by PBTS1, the situation is less clear. Although siderophore activity was detected (Figure 6E), none of the six NRPS-containing loci nor the 2 polyketide synthase (PKS) regions predicted by the antiSMASH analysis contained genes with homologies to siderophore-related functions (Supplementary Table S11). The antiSMASH analysis indicated that cluster 10 was 18% similar to a gene cluster of *Streptomyces* sp. AA4 involved in the production of the mixed-ligand siderophore amychelin (Seyedsayamdost et al., 2011), but the overlap was too low to predict this type of siderophore in PBTS1. Besides NRPS-based siderophore synthesis, a second mechanism termed NRPS-independent siderophore (NIS) biosynthesis is often deployed (Sheldon and Heinrichs, 2015), but BLAST searches with a variety of NIS-related genes from different organisms did not yield any significant hit with the PBTS1 chromosome. Therefore, the locus involved in the observed siderophore activity of this strain remains elusive.

Besides the capacity to produce their own siderophore, both PBTS1 and PBTS2 seem to be well-equipped to obtain siderophores from their environment (Supplementary Table S10). Indeed, PBTS1 has three siderophore-interacting proteins and three solitary iron-siderophore ABC transporter substrate-binding proteins, of which some might interact with the permeases of the ferric-chelate uptake system encoded in the PBTS1 chromosome. Additionally, this strain has a composite ABC transporter putatively involved in heme-iron acquisition, a putative heme binding protein, and a heme monooxygenase. Altogether, upon heme internalization, PBTS1 might be able to degrade it to release iron (Sheldon and Heinrichs, 2015). Similarly, PBTS2 contains two siderophore-interacting proteins, seven solitary iron-siderophore ABC transporter substrate-binding proteins, two ferric-chelate and one ferric-citrate uptake systems, and four iron acquisition composite ABC transporters. Although PBTS2 has one putative heme-iron uptake system, the heme-binding protein and the heme monooxygenase are absent. It thus appears that both PBTS isolates invest more energy in parasitizing on xeno-siderophores produced by other organisms, than in producing and secreting their own siderophores. This mode of action has been suggested to be more energy efficient and to provide a selective advantage over other bacteria in a heterogeneous population (Lemanceau et al., 2009; Sheldon and Heinrichs, 2015).

### PBTS1 and PBTS2 Have the Potential to Modulate the Hormone Homeostasis and the Development of Their Host

When the developmental changes that occur in “UCB-1” plants infected with the PBTS bacteria are analyzed, symptoms, such as the apical dominance loss and stunted growth of both shoots and roots, imply that the hormone status of the plant is strongly impacted upon colonization. In D188, the main virulence factor that affects plant development is the production of cytokinins through the linear plasmid-encoded *fas* pathway (Stes et al., 2011b). Additionally, basal levels of cytokinins are also produced by the chromosomally encoded tRNA degradation pathway (Francis et al., 2016), but the role of these cytokinins in the leafy gall pathology remains to be determined. Mining of the PBTS1 and PBTS2 chromosomes revealed the presence of an intact methylerythritol phosphate (MEP) pathway that provides the required cytokinin precursor molecule dimethylallyl pyrophosphate (DMAPP), a *miaA* and *miaB* gene encoding a tRNA-isopentenyltransferase and a tRNA methylthiolase, respectively, and a *LONELEY GUY* homolog encoding a phosphoribohydrolase activating cytokinin nucleotides (Supplementary Table S12). Thus, just as for the leafy gall inducer D188, the PBTS isolates are likely capable to produce cytokinins via the tRNA degradation pathway (Supplementary Figure S4). Although the relevance of this pathway for plant-interacting microbes has been questioned, given its omnipresence in microbes normally not associated with plants (Gray et al., 1996), more recent research has evidenced its importance. Indeed, tRNA-derived cytokinins have been shown to contribute positively to nodule development in *Aeschynomene* plants upon infection with *Bradyrhizobium* (Podlešáková et al., 2013) and to play a pivotal role in virulence of diverse fungi, including *Ustilago maydis* (Morrison et al., 2017), *Magnaporthe oryzae* (Chanclud et al., 2016), and *Claviceps purpurea* (Hinsch et al., 2016). In *Agrobacterium tumefaciens*, cytokinins, albeit not tRNA-derived, have been implicated in efficient attachment to plant tissues contributing to transformation efficiency (Sardesai et al., 2013), whereas in symbiosis, they are believed to have unexpectedly diverse and context-dependent functions, including mediating root susceptibility to rhizobial infection (Frugier et al., 2008). So, the different cytokinin levels produced by both pathways in D188 and probably also in both PBTS isolates (Figures 2–4 and Supplementary Figure S2), might have many more functionalities than the mere induction of shoots.

As the typical symptoms associated with an increased cytokinin response have been attributed to the degradation of auxin rather than the production of cytokinins in some leafy gall inducers (Lacey, 1948; Kemp, 1978), the chromosomes of PBTS1, PBTS2, and D188 were examined for the presence of homologs of the IAA catabolism (*iac*) genes implicated in auxin degradation in diverse bacteria (Leveau and Gerards, 2008), but none were detected. Despite this finding, we assessed whether the three strains could grow on IAA as sole carbon source. In agreement with the *in silico* prediction, PBTS1 and D188 did not proliferate on IAA, but, surprisingly, PBTS2 could (Table 4). Although the underlying catabolic pathway remains unknown, auxin degradation might contribute to symptom development in the PBTS disease.

**FIGURE 7:**
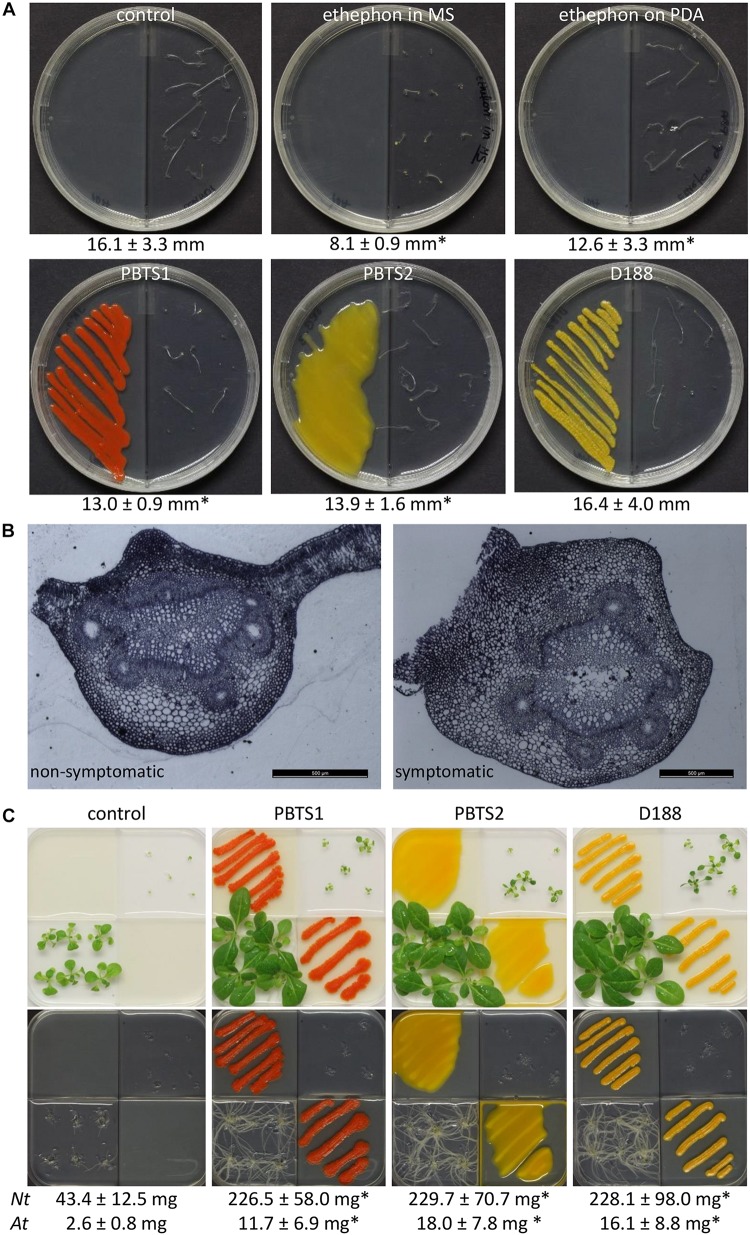
Effect of PBTS1 and PBTS2 on plant development. **(A)** Triple response assay used to evaluate ethylene production. PBTS1, PBTS2 and D188, as a reference, were inoculated on PDA medium in I-split plates and grown for 2 days at 28°C, then 10 *Arabidopsis* Col-0 seeds were placed in the adjacent sector on ½MS medium with 2% sucrose. PDA medium without any additions was used as negative control and 60 μM ethephon was either mixed in the ½MS medium or spotted on the PDA medium as positive controls. The length of the seedling hypocotyls (indicated below the images) was measured after 7 days and statistical differences compared to the untreated control were calculated with Student’s *t*-tests (^∗^significant difference at *P* < 0.05; *n* = 5–10). **(B)** Secondary differentiation of the vasculature of petioles of deformed leaves of “UCB-1” plants exhibiting PBTS symptoms compared to non-symptomatic leaves, suggesting an increased auxin response. **(C)** Production of plant growth promoting volatiles assessed in split plates on *Arabidopsis* Col-0 (right top quadrant) and *N. tabacum* W38 (left lower quadrant) grown on ½MS medium without sucrose. The bacteria were grown on PDA. After 11 days of growth the shoots were excised to determine their fresh weight (indicated below the images) and the root system was photographed. Statistical differences in the average fresh weight compared to the control were calculated with Student’s *t*-tests (^∗^significant difference at *P* < 0.01; *n* = 6 for *N. tabacum* (*Nt*); *n* = 5 for *Arabidopsis* (*At*)).

It was shown that D188 possibly modulates ethylene levels in its host by degrading the ethylene precursor 1-aminocyclopropane-1-carboxylic acid (ACC) via ACC deaminase activity (Francis et al., 2016). In the PBTS2 chromosome, but not that of PBTS1, an *acdS* gene flanked by its regulator *acdR* were identified (Supplementary Table S12), which is an accordance with the ability of PBTS2, but not PBTS1, to use ACC as a nitrogen source (Table 4). To further explore a possible role of ethylene in the interaction between the PBTS bacteria and plants, we carried out an *Arabidopsis* triple response assay in split plates (Guzmán and Ecker, 1990). When ethephon, used as the positive control, was mixed in the MS medium in which the *Arabidopsis* seeds germinated, a full triple response was obtained, i.e., shorter and thicker hypocotyls with an exaggerated apical hook (Figure 7A). However, when ethephon was applied on the PDA medium in the adjacent plate section, only an intermediate response was triggered, as illustrated by the significantly shorter hypocotyl length compared to the untreated control. Interestingly, both PBTS isolates provoked a similar intermediate response hinting at the production of low levels of ethylene, whereas D188 did not (Figure 7A). Bacterial ethylene production has been reported in several genera and it is generally accomplished by one of two pathways. Ethylene can either be spontaneously produced at trace amounts via oxidation of 2-keto-4-methylthiobutyric acid or it is generated from α-ketoglutarate and arginine by the ethylene-forming enzyme (EFE) (Eckert et al., 2014). Importantly, the non-enzymatic production of ethylene by *Pseudomonas solanacearum* is eliminated in the dark (Swanson et al., 1979). Assuming that this is also the case in the PBTS isolates, this would suggest that the observed ethylene production is enzyme-mediated. Nevertheless, because no significant hits were found with different EFE sequences from other microbes nor with ACC oxidase 1 enzyme (ACO1) sequences of plants, the pathway responsible for the putative ethylene production in the PBTS strains remains unresolved. In the symbiotic interaction between the legume *Sesbania rostrata* and *Azorhizobium caulinodans*, which is mediated via crack entry rather than via root hair colonization, plant-derived ethylene is required for rhizobial colonization. The combination of the bacterial Nod Factors and ethylene induces programmed cell death that allows the intercellular progression of the bacteria through the cortex toward the developing nodule primordium (D’Haeze et al., 2003; Guinel, 2015). Additionally, in the relatively recently evolved symbiosis between the non-legume *Parasponia andersonii* and rhizobia such as *Bradyrhizobium elkanii*, ethylene is also required for endophytic infection (Seifi Kalhor et al., 2016). Even more, in *Pseudomonas syringae* pv. *glycinea*, *efe* mutants were significantly impaired in their ability to grow *in planta* (Weingart et al., 2001). Thus, we speculate that the putative bacterium-derived ethylene plays a role in the endophytic colonization process. Alternatively, ethylene production by soil microorganisms has been shown to affect the viability of other microbes (Smith and Cook, 1974), so a role in niche protection could also be envisioned.

ACC deaminase activity is often detected in bacteria that produce auxins (Glick, 2014). Strain D188 produces auxin via a chromosome-encoded indole-pyruvate pathway in which tryptophan serves as a precursor (Vandeputte et al., 2005), and accordingly, a putative indole-pyruvate decarboxylase *ipdC* gene has been identified in its chromosome (*A3L23_RS23370*) (Francis et al., 2016). The much more efficient IAA production when tryptophol (indole-3-ethanol) is fed as a precursor to D188, signifies that the IpdC activity represents the rate-limiting step in auxin production (Vandeputte et al., 2005). In D188, auxin is believed to play a crucial role in the increased auxin signaling that is at the basis of the neovascularization of the developing leafy gall (Dolzblasz et al., 2018). Interestingly, light microscopy analysis of petioles of control and symptomatic “UCB-1” leaves, clearly showed thickening of the tissue and aberrations in the vasculature (Figure 7B), that are highly reminiscent of those observed upon infection of *Arabidopsis* with D188 (Dolzblasz et al., 2018), implying the putative involvement of auxin in the PBTS symptomatology. In this respect, in the PBTS1 and PBTS2 chromosomes, all the genes of a tryptophan biosynthesis pathway similar to that of D188 were identified as well as a gene homologous to *A3L23_RS23370* (Supplementary Table S12 and Supplementary Figure S5).

Despite the weak homology of A3L23_RS23370 to the IpdC of *Paenibacillus polymyxa* (31% identity, 47% similarity) (Phi et al., 2008) and its ability to increase IAA production in *Escherichia coli* (Francis et al., 2016), the protein is annotated as acetolactate synthase. In the genome of *P. polymyxa*, two homologs of this gene are present: PPSC2_RS39450, which is the IpdC, and PPSC2_RS35795, annotated as a biosynthetic type of acetolactate synthase (large subunit); both proteins are 30% identical and 48% similar. Downstream of the latter, *PPSC2_RS35800* encodes an acetolactate synthase (small subunit) followed by two genes, *PPSC2_RS35810* and *PPSC2_RS35815*, involved in leucine biosynthesis (Ma et al., 2011). In the D188 and PBTS chromosomes, only one acetolactate synthase gene is present and the encoded protein has a slightly higher homology to PPSC2_RS35795 (50% identity, 66% similarity) than to the IpdC. Additionally, in all three chromosomes, an acetolactate synthase (small subunit) gene and two genes involved in branched-chain amino acid biosynthesis occur immediately downstream of this gene (Supplementary Table S12), an organization that is very similar to that of the second gene cluster in *P. polymyxa*. Thus, the *ipdC*-like gene in the *Rhodococcus* chromosomes might function in branched-chain amino acid biosynthesis rather than in auxin biosynthesis (Supplementary Figure S5). Nevertheless, because the only copy of this gene present in the PBTS bacteria and in D188 has a low homology level to both of the proteins in *P. polymyxa* and since no other genes putatively related to auxin biosynthesis appear to be present in the *Rhodococcus* chromosomes, we speculate that this IpdC-like protein might function in the two pathways by utilizing both pyruvate and indole-pyruvate as substrates. Recently, several indole-3-acetaldehyde dehydrogenases have been identified in *Pseudomonas syringae* that convert indole-3-acetaldehyde into IAA (McClerklin et al., 2018). In the three *Rhodococcus* strains, homologs are found to PSPTO_0092, the enzyme with the highest activity in *P. syringae* (Supplementary Table S12), that possibly encode the last step in IAA production via the indole-pyruvic acid pathway (Supplementary Figure S5). To validate the prediction that PBTS1 and PBTS2 can produce auxin, the bacteria were grown in the presence of tryptophan and tryptophol, the two auxin precursors that are used by strain D188 with different efficiencies (Vandeputte et al., 2005), and the occurrence of indolic compounds was evaluated using Salkowski’s reagent (Glickman and Dessaux, 1995). When PBTS1, PBTS2, and D188 were grown in YEB medium supplemented with either of the precursors, no auxin could be detected with the colorimetric assay in any of the supernatants (Table 5). In contrast, detectable auxin production by the three *Rhodococcus* strains could be demonstrated when tryptophan, but especially tryptophol, was provided, in line with previous findings on auxin production by D188 (Vandeputte et al., 2005). In the presence of tryptophol, PBTS2 and D188 produced a comparable level of auxin, which was about 5 times higher than that measured for PBTS1 under the experimental conditions used (Table 5). Altogether, in analogy with strain D188, these data suggest that PBTS1 and PBTS2 might produce auxin via the indole-pyruvate pathway with the IpdC activity representing the rate limiting step.

**TABLE 5:** Production of IAA by PBTS1, PBTS2, and D188 under different growth conditions.

	μg IAA/OD_600_		
	PBTS1	PBTS2	D188
YEB + tryptophan	0.19 ± 0.02	0.26 ± 0.12	0.25 ± 0.03
YEB + tryptophol	0.16 ± 0.03	0.32 ± 0.07	0.36 ± 0.08
JM + tryptophan	2.67 ± 0.04	3.71 ± 0.35	5.47 ± 0.37
JM + tryptophol	5.12 ± 1.34	25.20 ± 0.59	23.28 ± 4.24

**The data are the average of three independent experiments.**

Besides its role as an intermediate in the biosynthesis of branched-chain amino acids, acetolactate can spontaneously convert to diacetyl that can be used by an acetoin reductase to form acetoin (3-hydroxy-2-butanone), that is further transformed into 2,3-butanediol by a 2,3-butanediol dehydrogenase, a common bacterial pathway known as butanediol fermentation (Xiao and Xu, 2007). Interestingly, the latter two compounds have been identified as important volatiles produced by beneficial bacteria that exhibit significant plant growth-promoting effects and trigger induced systemic resistance (Ryu et al., 2005). Because of the presence of the two genes required for butanediol fermentation in the three *Rhodococcus* chromosomes (Supplementary Table S12 and Supplementary Figure S5), we evaluated whether the production of plant growth-promoting volatiles could be demonstrated in a plate assay with both *Arabidopsis* and *N. tabacum* as test plants. A strong biostimulating effect on the shoots as well as the roots of both plants was observed upon exposure to the volatiles of the three bacteria (Figure 7C). The PBTS bacteria have been postulated to be beneficial rather than pathogenic (Savory et al., 2017). However, in contrast to the effect of the plasmid-free D188 derivative (Francis et al., 2016), no positive impact on plant development was ever observed under our experimental conditions when the PBTS bacteria were in contact with their host (Figure 2). Furthermore, the clear plant growth-promoting effect accomplished by the volatiles of the PBTS isolates, should not be considered as a proof of their alleged beneficial character because many notorious phytopathogens have been shown to produce volatiles that positively affect plants (Sánchez-López et al., 2016). Eventually, a function in niche occupation can be envisioned, since 2,3-butanediol has recently been shown to be important for rhizosphere robustness in the rhizobacterium *Bacillus subtilis* (Yi et al., 2016).

## Conclusion

By combining previous results (Stamler et al., 2015b) with the data presented here, we conclude that subpopulations of the PBTS bacteria carry a *fas* locus that is highly similar to that present in leafy gall inducers. Additionally, for PBTS2, the *fas* operon is probably present on a linear plasmid, but for PBTS1, not enough data are currently available to support the same hypothesis. In contrast to many leafy gall inducers, the elusive virulence carrier in the two PBTS strains is highly unstable outside the plant host, affecting the outcome of pathogenicity assays. Nevertheless, the chromosomes of the PBTS strains encode multiple functions that could contribute to the interaction with and developmental modification of the plant host, including those implicated in the modulation of auxin and ethylene levels and in the production of cytokinins and growth-affecting volatiles. These findings suggest that, similar to leafy gall inducers, co-option between the virulence carrier and the chromosome is likely an important factor in the virulence strategy of these bacteria.

Our data also revealed that the co-occurrence of PBTS1 and PBTS2 is supported by a diversification in nutrient utilization patterns and in complementary activities that could stimulate niche partitioning. PBTS1 would strongly benefit from the presence of PBTS2 to occupy habitats that could otherwise be too hostile for this strain. In this context, it is tempting to speculate on the origin of PBTS: by associating with the pathogenic PBTS2, PBTS1, as an initially harmless inhabitant of pistachio, found itself in a situation permissive for virulence gene exchange which resulted in synergistic activities that strongly impacted the development of their host. Until the virulence carriers of PBTS1 and PBTS2 are identified and thoroughly analyzed, this scenario cannot be validated, but the chromosome analysis presented here allows to get a first glance at the mode of action of these recently emerged pathogens.

## Data Availability Statement

The raw data supporting the conclusions of this article will be made available by the authors, without undue reservation, to any qualified researcher.

## Author Contributions

DV, IF, RS, JK, and JR designed the experimental work. DV, IF, PL, JV, RS, JK, and JR performed the experiments and analyzed the data. YZ performed the bioinformatics analysis. DV wrote the paper. All authors critically read the manuscript and approved its final version.

## Conflict of Interest

The authors declare that the research was conducted in the absence of any commercial or financial relationships that could be construed as a potential conflict of interest.

## Footnotes

1 https://crisprcas.i2bc.paris-saclay.fr/CrisprCasFinder/Index

2 https://github.com/widdowquinn/scripts/blob/master/bioinformatics/calculate_ani.py

3 https://ggdc.dsmz.de/ggdc.php#

4 ftp://ftp.ncbi.nih.gov/pub/COG/COG/myva

5 http://www.kegg.jp/blastkoala/

6 www.kegg.jp/kegg/tool/map_pathway.html

7 http://rrndb.umms.med.umich.edu/

## References

[B1] Achari G. A. Ramesh R. (2014). Diversity, biocontrol, and plant growth promoting abilities of xylem residing bacteria from solanaceous crops. *Int. J. Microbiol.* 2014:14. 10.1155/2014/296521 24963298 PMC4055287

[B2] Al Akhrass F. Al Wohoush I. Chaftari A.-M. Reitzel R. Jiang Y. Ghannoum M. (2012). *Rhodococcus bacteremia* in cancer patients is mostly catheter related and associated with biofilm formation. *PLoS One* 7:e32945. 10.1371/journal.pone.0032945 22427914 PMC3302794

[B3] Alvarez H. M. (2019). *Biology of Rhodococcus (Microbiology Monographs, 16).* Cham: Springer Publishing International.

[B4] Anastasi E. MacArthur I. Scortti M. Alvarez S. Giguère S. Vázquez-Boland J. A. (2016). Pangenome and phylogenomic analysis of the pathogenic actinobacterium *Rhodococcus equi*. *Genome Biol. Evol.* 8 3140–3148. 10.1093/gbe/evw222 27638249 PMC5174736

[B5] Barka E. A. Vatsa P. Sanchez L. Gaveau-Vaillant N. Jacquard C. Klenk H.-P. (2016). Taxonomy, physiology, and natural products of Actinobacteria. *Microbiol. Mol. Biol. Rev.* 80 1–43. 10.1128/MMBR.00019-15 26609051 PMC4711186

[B6] Barreca D. Laganà G. Leuzzi U. Smeriglio A. Trombetta D. Bellocco E. (2016). Evaluation of the nutraceutical, antioxidant and cytoprotective properties of ripe pistachio (*Pistacia vera* L., variety Bronte) hulls. *Food Chem.* 196 493–502. 10.1016/j.foodchem.2015.09.077 26593519

[B7] Bayat Z. Hassanshahian M. Cappello S. (2015). Immobilization of microbes for bioremediation of crude oil polluted environments: a mini review. *Open Microbiol. J.* 9 48–54. 10.2174/1874285801509010048 26668662 PMC4676050

[B8] Belimov A. A. Dodd I. C. Safronova V. I. Dumova V. A. Shaposhnikov A. I. Ladatko A. G. (2014). Abscisic acid metabolizing rhizobacteria decrease ABA concentrations in planta and alter plant growth. *Plant Physiol. Biochem.* 74 84–91. 10.1016/j.plaphy.2013.10.032 24270514

[B9] Blin K. Shaw S. Steinke K. Villebro R. Ziemert N. Lee S. Y. (2019). antiSMASH 5.0: updates to the secondary metabolite genome mining pipeline. *Nucleic Acids Res.* 47 W81–W87. 10.1093/nar/gkz310 31032519 PMC6602434

[B10] Bucher T. Keren-Paz A. Hausser J. Olender T. Cytryn E. Kolodkin-Gal I. (2019). An active β-lactamase is a part of an orchestrated cell wall stress resistance network of Bacillus subtilis and related rhizosphere species. *Environ. Microbiol.* 21 1068–1085. 10.1111/1462-2920.14526 30637927

[B11] Butterworth D. Cole M. Hanscomb G. Rolinson G. N. (1979). Olivanic acids, a family of β-lactam antibiotics with β-lactamase inhibitory properties produced by *Streptomyces* species. *J. Antibiotics* 32 287–294. 10.7164/antibiotics.32.287 468715

[B12] Ceniceros A. Dijkhuizen L. Petrusma M. Medema M. H. (2017). Genome-based exploration of the specialized metabolic capacities of the genus *Rhodococcus*. *BMC Genomics* 18:593. 10.1186/s12864-017-3966-3961 28793878 PMC5550956

[B13] Chanclud E. Kisiala A. Emery N. R. J. Chalvon V. Ducasse A. Romiti-Michel C. (2016). Cytokinin production by the rice blast fungus is a pivotal requirement for full virulence. *PLoS Pathog.* 12:e1005457. 10.1371/journal.ppat.1005457 26900703 PMC4765853

[B14] Couvin D. Bernheim A. Toffano-Nioche C. Touchon M. Michalik J. Néron B. (2018). CRISPRCasFinder, an update of CRISRFinder, includes a portable version, enhanced performance and integrates search for Cas proteins. *Nucleic Acids Res.* 46 W246–W251. 10.1093/nar/gky425 29790974 PMC6030898

[B15] Creason A. L. Vandeputte O. M. Savory E. A. Davis E. W. II Putnam M. L. Hu E. (2014). Analysis of genome sequences from plant pathogenic *Rhodococcus* reveals genetic novelties in virulence loci. *PLoS One* 9:e101996. 10.1371/journal.pone.0101996 25010934 PMC4092121

[B16] Crespi M. Messens E. Caplan A. B. Van Montagu M. Desomer J. (1992). Fasciation induction by the phytopathogen *Rhodococcus fascians* depends upon a linear plasmid encoding a cytokinin synthase gene. *EMBO J.* 11 795–804. 10.1002/j.1460-2075.1992.tb05116.x 1547783 PMC556518

[B17] Darling A. C. E. Mau B. Blattner F. R. Perna N. T. (2004). Mauve: multiple alignment of conserved genomic sequence with rearrangements. *Genome Res.* 14 1394–1403. 10.1101/gr.2289704 15231754 PMC442156

[B18] De Voss J. J. Rutter K. Schroeder B. G. Su H. Zhu Y. Barry C. E. III (2000). The salicylate-derived mycobactin siderophores of *Mycobacterium tuberculosis* are essential for growth in macrophages. *Proc. Natl. Acad. Sci. U.S.A.* 97 1252–1257. 10.1073/pnas.97.3.1252 10655517 PMC15586

[B19] DeLorenzo D. M. Moon T. S. (2018). Selection of stable reference genes for RT-qPCR in *Rhodococcus opacus* PD630. *Sci. Rep.* 8:6019. 10.1038/s41598-018-24486-w 29662144 PMC5902447

[B20] Depuydt S. De Veylder L. Holsters M. Vereecke D. (2009a). Eternal youth, the fate of developing *Arabidopsis* leaves upon *Rhodococcus fascians* infection. *Plant Physiol.* 149 1387–1398. 10.1104/pp.108.131797 19118126 PMC2649406

[B21] Depuydt S. Trenkamp S. Fernie A. R. Elftieh S. Renou J.-P. Vuylsteke M. (2009b). An integrated genomics approach to define niche establishment by *Rhodococcus fascians*. *Plant Physiol.* 149 1366–1386. 10.1104/pp.108.131805 19118125 PMC2649413

[B22] Depuydt S. Doležal K. Van Lijsebettens M. Moritz T. Holsters M. Vereecke D. (2008a). Modulation of the hormone setting by *Rhodococcus fascians* results in ectopic KNOX activation in *Arabidopsis*. *Plant Physiol.* 146 1267–1281. 10.1104/pp.107.113969 18184732 PMC2259056

[B23] Depuydt S. Putnam M. Holsters M. Vereecke D. (2008b). “*Rhodococcus fascians*, an emerging threat for ornamental crops,” in *Floriculture, Ornamental, and Plant Biotechnology: Advances and Topical Issues*, Vol. 5 ed. Teixeira da Silva J. A., (Isleworth: Global Science Books), 480–489.

[B24] Desomer J. Dhaese P. Van Montagu M. (1988). Conjugative transfer of cadmium resistance plasmids in *Rhodococcus fascians* strains. *J. Bacteriol.* 170 2401–2405. 10.1128/jb.170.5.2401-2405.1988 3162908 PMC211139

[B25] D’Haeze W. De Rycke R. Mathis R. Goormachtig S. Pagnotta S. Verplancke C. (2003). Reactive oxygen species and ethylene play a positive role in lateral root base nodulation of a semiaquatic legume. *Proc. Natl. Acad. Sci. U.S.A.* 100 11789–11794. 10.1073/pnas.1333899100 12975522 PMC208836

[B26] Dhandapani P. Song J. Novak O. Jameson P. E. (2017). Infection by *Rhodococcus fascians* maintains cotyledons as a sink tissue for the pathogen. *Ann. Bot.* 119 841–852. 10.1093/aob/mcw202 27864224 PMC5378184

[B27] Dolzblasz A. Banasiak A. Vereecke D. (2018). Neovascularization during leafy gall formation on *Arabidopsis* thaliana upon *Rhodococcus fascians* infection. *Planta* 247 215–228. 10.1007/s00425-017-2778-2775 28942496

[B28] Eckert C. Xu W. Xiong W. Lynch S. Ungerer J. Tao L. (2014). Ethylene-forming enzyme and bioethylene production. *Biotechnol. Biofuels* 7:33. 10.1186/1754-6834-7-33 24589138 PMC3946592

[B29] Elsayed Y. Refaat J. Abdelmohsen U. R. Fouad M. A. (2017). The genus *Rhodococcus* as a source of novel bioactive substances: a review. *J. Pharmacogn. Phytochem.* 6 83–92.

[B30] Faure D. Vereecke D. Leveau J. H. J. (2009). Molecular communication in the rhizosphere. *Plant Soil* 321 279–303. 10.1007/s11104-008-9839-9832

[B31] Francis I. De Keyser A. De Backer P. Simón-Mateo S. Kalkus K. Pertry I. (2012). pFiD188, the linear virulence plasmid of *Rhodococcus fascians* strain D188. *Mol. Plant-Microbe Interact.* 25 637–647. 10.1094/MPMI-08-11-0215 22482837

[B32] Francis I. M. Stes E. Zhang Y. Rangel D. Audenaert K. Vereecke D. (2016). Mining the genome of *Rhodococcus fascians*, a plant growth-promoting bacterium gone astray. *New Biotechnol.* 33 706–717. 10.1016/j.nbt.2016.01.009 26877150

[B33] Francis I. M. Vereecke D. (2019). “Plant-associated *Rhodococcus* species, for better and for worse,” in *Biology of Rhodococcus, (Microbiology Monographs, 16)*, 2nd Edn, ed. Alvarez H. M., (Cham: Springer International Publishing), 359–377. 10.1007/978-3-030-11461-9_13

[B34] Frugier F. Kosuta S. Murray J. D. Crespi M. Szczyglowski K. (2008). Cytokinin: secret agent of symbiosis. *Trends Plant Sci.* 13 115–120. 10.1016/j.tplants.2008.01.003 18296104

[B35] Glick B. R. (2014). Bacteria with ACC deaminase can promote plant growth and help to feed the world. *Microbiol. Res.* 169 30–39. 10.1016/j.micres.2013.09.009 24095256

[B36] Glickman E. Dessaux Y. (1995). A critical examination of the specificity of the Salkowski reagent for indolic compounds produced by phytopathogenic bacteria. *Appl. Environ. Microbiol.* 61 793–796. 10.1128/aem.61.2.793-796.1995 16534942 PMC1388360

[B37] Gray J. Gelvin S. B. Meilan R. Morris R. O. (1996). Transfer RNA is the source of extracellular isopentenyladenine in a Ti-plasmidless strain of *Agrobacterium tumefaciens*. *Plant Physiol.* 110 431–438. 10.1104/pp.110.2.431 12226194 PMC157737

[B38] Guinel F. C. (2015). Ethylene, a hormone at the center-stage of nodulation. *Front. Plant Sci.* 6:1121. 10.3389/fpls.2015.01121 26834752 PMC4714629

[B39] Gupta N. Skinner K. A. Summers Z. M. Edirisinghe J. N. Faria J. P. Marshall C. W. (2019). Draft genome sequence of *Rhodococcus* sp. strain ATCC 49988, a quinoline-degrading bacterium. *Microbiol. Resour. Announc.* 8 e00403–e00419. 10.1128/MRA.00403-419 31221646 PMC6588367

[B40] Guzmán P. Ecker J. R. (1990). Exploiting the triple response of Arabidopsis to identify ethylene-related mutants. *Plant Cell* 2 513–523. 10.1105/tpc.2.6.513 2152173 PMC159907

[B41] Hassen A. Saidi N. Cherif M. Boudabous A. (1998). Resistance of environmental bacteria to heavy metals. *Bioresour. Technol.* 64 7–15. 10.1016/S0960-8524(97)00161-162

[B42] He Y.-H. Isono S. Shibuya M. Tsuji M. Purushothama C.-R. A. Tanaka K. (2012). Oligo-DNA custom macroarray for monitoring major pathogenic and non-pathogenic fungi and bacteria in the phyllosphere of apple trees. *PLoS One* 7:e34249. 10.1371/journal.pone.0034249 22479577 PMC3316626

[B43] Hinsch J. Galuszka P. Tudzynski P. (2016). Functional characterization of the first filamentous fungal tRNA-isopentenyltransferase and its role in the virulence of *Claviceps purpurea*. *New Phytol.* 211 980–992. 10.1111/nph.13960 27074411

[B44] Jacobs S. E. Mohanty U. (1951). Studies in bacteriosis XXVII. Factors influencing infection by *Corynebacterium fascians* (Tilford) Dowson. *Ann. Appl. Biol.* 38 237–245. 10.1111/j.1744-7348.1951.tb07800.x

[B45] Jameson P. E. Dhandapani P. Song J. Zatloukal M. Strnad M. Remus-Emsermann M. N. P. (2019). The cytokinin complex associated with *Rhodococcus fascians*: which compounds are critical for virulence? *Front. Plant Sci.* 10:674. 10.3389/fpls.2019.00674 31191583 PMC6539147

[B46] Kado C. I. Heskett M. G. (1970). Selective media for *Agrobacterium*, *Corynebacterium*, *Erwinia* and *Xanthomonas*. *Phytopathology* 60 969–976. 10.1094/Phyto-60-969 5469886

[B47] Kallsen C. E. Holtz B. Villaruz L. Wylie C. (2000). Leaf zinc and copper concentrations of mature pistachio trees in response to fertigation. *HortTechnology* 10 172–176. 10.21273/HORTTECH.10.1.172

[B48] Kanehisa M. Sato Y. Morishima K. (2016). BlastKOALA and GhostKOALA: KEGG tools for functional characterization of genome and metagenome sequences. *J. Mol. Biol.* 428 726–731. 10.1016/j.jmb.2015.11.006 26585406

[B49] Kemp D. R. (1978). “Indole-3-ylacetic acid metabolism of *Corynebacterium fascians*,” in *Microbial Ecology*, eds Loutit M. W. Miles J. A. R., (Berlin: Springer-Verlag), 341–345. 10.1007/978-3-642-67034-3_67

[B50] Khalil N. Corker L. Powell E. A. Mortensen J. E. (2019). Neonatal bacteremia and oligoarthritis caused by *Rhodococcus corynebacterioides*/*Rhodococcus kroppenstedtii*. *Diagn. Microbiol. Infect. Dis.* 94 395–397. 10.1016/j.diagmicrobio.2019.02.005 30857916

[B51] Kitamura Y. Sawabe E. Ohkusu K. Tojo N. Tohda S. (2012). First report of sepsis caused by *Rhodococcus corynebacterioides* in a patient with myelodysplastic syndrome. *J. Clin. Microbiol.* 50 1089–1091. 10.1128/jcm.06279-6211 22205796 PMC3295164

[B52] Krokene P. Solheim H. (2001). Loss of pathogenicity in the blue-stain fungus *Ceratocystis polonica*. *Plant Pathol.* 50 497–502. 10.1046/j.1365-3059.2001.00588.x

[B53] Lacey M. S. (1948). Studies on Bacterium fascians. Part V. Further observations on the pathological and physiological reactions of Bact. fascians. *Ann. Appl. Biol.* 35 572–581. 10.1111/j.1744-7348.1948.tb07399.x

[B54] Lee L.-H. Chan K.-G. Stach J. Wellington E. M. H. Goh B.-H. (2018). *The Search for Biological Active Agent(s) From Actinobacteria.* Lausanne: Frontiers Media. 10.3389/fmicb.2018.00824 PMC5946001 29780365

[B55] Lee Z. M.-P. Bussema C. III Schmidt T. M. (2009). rrnDB: documenting the number of rRNA and tRNA genes in bacteria and archaea. *Nucleic Acids Res.* 37 D489–D493. 10.1093/nar/gkn689 18948294 PMC2686494

[B56] Lemanceau P. Expert D. Gaymard F. Bakker P. A. H. M. Briat J.-F. (2009). “Role of iron in plant-microbe interactions,” in *Plant Innate Immunity*, 1st Edn, ed. Van Loon L. J. C., (London: Academic Press Ltd), 491–549.

[B57] Leveau J. H. J. Gerards S. (2008). Discovery of a bacterial gene cluster for catabolism of the plant hormone indole 3-acetic acid. *FEMS Microbiol. Ecol.* 65 238–250. 10.1111/j.1574-6941.2008.00436.x 18205812

[B58] Li L. Stoeckert C. J. J. Roos D. S. (2003). OrthoMCL: identification of ortholog groups for eukaryotic genomes. *Genome Res.* 13 2178–2189. 10.1101/gr.1224503 12952885 PMC403725

[B59] Ma M. Wang C. Ding Y. Li L. Shen D. Jiang X. (2011). Complete genome sequence of *Paenibacillus polymyxa* SC2, a strain of plant growth-promoting rhizobacterium with broad-spectrum antimicrobial activity. *J. Bacteriol.* 193 311–312. 10.1128/jb.01234-1210 21037012 PMC3019932

[B60] Maes T. Vereecke D. Ritsema T. Cornelis K. Ngo T. T. H. Van Montagu M. (2001). The att locus of *Rhodococcus fascians* strain D188 is essential for full virulence on tobacco through the production of an autoregulatory compound. *Mol. Microbiol.* 42 13–28. 10.1046/j.1365-2958.2001.02615.x 11679063

[B61] McClerklin S. A. Lee S. G. Harper C. P. Nwumeh R. Jez J. M. Kunkel B. N. (2018). Indole-3-acetaldehyde dehydrogenase-dependent auxin synthesis contributes to virulence of *Pseudomonas syringae* strain DC3000. *PLoS Pathog.* 14:e1006811. 10.1371/journal.ppat.1006811 29293681 PMC5766252

[B62] Meier-Kolthoff J. P. Auch A. F. Klenk H.-P. Göker M. (2013). Genome sequence-based species delimitation with confidence intervals and improved distance functions. *BMC Bioinformatics* 14:60. 10.1186/1471-2105-14-60 23432962 PMC3665452

[B63] Michailides T. J. (2005). “Above ground fungal diseases,” in *Pistachio Production Manual*, 4th Edn, eds Ferguson L. Beede R. H. Freeman M. W. Haviland D. R. Holtz B. A. Kallsen C. E., (Davis: Fruit and Nut Research and Information Center), 214–232.

[B64] Miller J. H. (1972). *Experiments in Molecular Genetics.* New York, NY: Cold Spring Harbor Laboratory.

[B65] Mitra P. (2015). Pathogenicity of *Leishmania donovani* is associated with the high expression of a group low molecular weight proteins. *Trop. Parasitol.* 5 106–117. 10.4103/2229-5070.162521 26629453 PMC4557149

[B66] Morrison E. N. Emery R. J. N. Saville B. J. (2017). Fungal derived cytokinins are necessary for normal *Ustilago maydis* infection of maize. *Plant Pathol.* 66 726–742. 10.1111/ppa.12629

[B67] Nikolaeva E. V. Kang S. Olson T. N. Kim S. H. (2012). Real-time PCR detection of *Rhodococcus fascians* and discovery of new plants associated with *R. fascians* in Pennsylvania. *Plant Health Prog.* 13:24 10.1094/PHP-2012-0227-02-RS

[B68] Nikolaeva E. V. Park S.-Y. Kang S. Olson T. N. Kim S. H. (2009). Ratios of cells with and without virulence genes in *Rhodococcus fascians* populations correlate with degrees of symptom development. *Plant Dis.* 93 499–506. 10.1094/PDIS-93-5-0499 30764134

[B69] Pan L. Gu J.-G. Yin B. Cheng S.-P. (2009). Contribution to deterioration of polymeric materials by a slow-growing bacterium *Nocardia corynebacterioides*. *Int. Biodeterior. Biodegrad.* 63 24–29. 10.1016/j.ibiod.2008.06.003

[B70] Pertry I. Václavíková K. Depuydt S. Galuszka P. Spíchal L. Temmerman W. (2009). Identification of *Rhodococcus fascians* cytokinins and their modus operandi to reshape the plant. *Proc. Natl. Acad. Sci. U.S.A.* 106 929–934. 10.1073/pnas.0811683106 19129491 PMC2630087

[B71] Pertry I. Václavíková K. Gemrotová M. Spíchal L. Galuszka P. Depuydt S. (2010). *Rhodococcus fascians* impacts plant development through the dynamic Fas-mediated production of a cytokinin mix. *Mol. Plant-Microbe Interact.* 23 1164–1174. 10.1094/MPMI-23-9-1164 20687806

[B72] Phi Q.-T. Park Y.-M. Ryu C.-M. Park S.-H. Ghim S.-Y. (2008). Functional identification and expression of indole-3-pyruvate decarboxylase from *Paenibacillus polymyxa* E681. *J. Microbiol. Biotechnol.* 18 1235–1244. 18667851

[B73] Podlešáková K. Fardoux J. Patrel D. Bonaldi K. Novák O. Strnad M. (2013). Rhizobial synthesized cytokinins contribute to but are not essential for the symbiotic interaction between photosynthetic bradyrhizobia and *Aeschynomene legumes*. *Mol. Plant-Microbe Interact.* 26 1232–1238. 10.1094/mpmi-03-13-0076-r 23777431

[B74] Putnam M. L. Miller M. L. (2007). *Rhodococcus fascians* in herbaceous perennials. *Plant Dis.* 91 1064–1076. 10.1094/PDIS-91-9-1064 30780643

[B75] Radhika V. Ueda N. Tsuboi Y. Kojima M. Kikuchi J. Kudo T. (2015). Methylated cytokinins from the phytopathogen *Rhodococcus fascians* mimic plant hormone activity. *Plant Physiol.* 169 1118–1126. 10.1104/pp.15.00787 26251309 PMC4587462

[B76] Randall J. J. Stamler R. A. Kallsen C. E. Fichtner E. J. Heerema R. J. Cooke P. (2018). Comment on “Evolutionary transitions between beneficial and phytopathogenic *Rhodococcus* challenge disease management”. *eLife* 7:e35272. 10.7554/eLife.35272 29737967 PMC5951677

[B77] Reddy P. V. Puri R. V. Chauhan P. Kar R. Rohilla A. Khera A. (2013). Disruption of mycobactin biosynthesis leads to attenuation of *Mycobacterium tuberculosis* for growth and virulence. *J. Infect. Dis.* 208 1255–1265. 10.1093/infdis/jit250 23788726

[B78] Risa A. Krifaton C. Kukolya J. Kriszt B. Cserháti M. Táncsics A. (2018). Aflatoxin B1 and zearalenone-detoxifying profile of *Rhodococcus* type strains. *Curr. Microbiol.* 75 907–917. 10.1007/s00284-018-1465-1465 29511873

[B79] Ryu C.-M. Farag M. A. Paré P. W. Kloepper J. (2005). Invisible signals from the underground: bacterial volatiles elicit plant growth promotion and induce systemic resistance. *Plant Pathol. J.* 21 7–12. 10.5423/PPJ.2005.21.1.007

[B80] Sabart P. R. Gakovich D. Hanson R. S. (1986). Avirulent isolates of *Corynebacterium fascians* that are unable to utilize agmatine and proline. *Appl. Environ. Microbiol.* 52 33–36. 10.1128/aem.52.1.33-36.1986 3729405 PMC203388

[B81] Sánchez-López A. M. Baslam M. De Diego N. Muñoz F. J. Bahaji A. Almagro G. (2016). Volatile compounds emitted by diverse phytopathogenic microorganisms promote plant growth and flowering through cytokinin action. *Plant Cell Environ.* 39 2592–2608. 10.1111/pce.12759 27092473

[B82] Sangal V. Goodfellow M. Jones A. L. Schwalbe E. C. Blom J. Hoskisson P. A. (2016). Next-generation systematics: an innovative approach to resolve the structure of complex prokaryotic taxa. *Sci. Rep.* 6:38392. 10.1038/srep38392 27924912 PMC5141411

[B83] Sangal V. Goodfellow M. Jones A. L. Seviour R. J. Sutcliffe I. C. (2019). “Refined systematics of the genus *Rhodococcus* based on whole genome analyses,” in *Biology of Rhodococcus, (Microbiology Monographs, 16)*, 2nd Edn, ed. Alvarez H. M., (Cham: Springer International Publishing), 1–21. 10.1007/978-3-030-11461-9_1

[B84] Sardesai N. Lee L.-Y. Chen H. Yi H. Olbricht G. R. Stirnberg A. (2013). Cytokinins secreted by *Agrobacterium* promote transformation by repressing a plant Myb transcription factor. *Sci. Signal.* 6:ra100. 10.1126/scisignal.2004518 24255177

[B85] Savory E. A. Fuller S. L. Weisberg A. J. Thomas W. J. Gordon M. I. Stevens D. M. (2017). Evolutionary transitions between beneficial and phytopathogenic *Rhodococcus* challenge disease management. *eLife* 6:e30925. 10.7554/eLife.30925 29231813 PMC5726852

[B86] Schlechter R. O. Miebach M. Remus-Emsermann M. N. P. (2019). Driving factors of epiphytic bacterial communities: a review. *J. Adv. Res.* 19 57–65. 10.1016/j.jare.2019.03.003 31341670 PMC6630024

[B87] Scott J. C. Greenhut I. V. Leveau J. H. J. (2013). Functional characterization of the bacterial iac genes for degradation of the plant hormone indole-3-acetic acid. *J. Chem. Ecol.* 39 942–951. 10.1007/s10886-013-0324-x 23881445

[B88] Seifi Kalhor M. Holmer R. van Zeijl A. Bisseling T. Geurts R. (2016). “Dual effect of ethylene on root nodulation of *Parasponia andersonii*,” in *Genetic Constraints that Determine Rhizobium-Root Nodule Formation in Parasponia andersonii, PhD Thesis*, ed. Seifi Kalhor M., (Wageningen: Wageningen University), 105–121. 10.18174/388906

[B89] Seyedsayamdost M. R. Traxler M. F. Zheng S.-L. Kolter R. Clardy J. (2011). Structure and biosynthesis of amychelin, an unusual mixed-ligand siderophore from *Amycolatopsis* sp. AA4. *J. Am. Chem. Soc.* 133 11434–11437. 10.1021/ja203577e 21699219 PMC3144690

[B90] Sheldon J. R. Heinrichs D. E. (2015). Recent developments in understanding the iron acquisition strategies of gram positive pathogens. *FEMS Microl. Rev.* 39 592–630. 10.1093/femsre/fuv009 25862688

[B91] Smith A. M. Cook R. J. (1974). Implications of ethylene production by bacteria for biological balance of soil. *Nature* 252 703–705. 10.1038/252703b04437621

[B92] Soliemanzadeh A. Mozafari V. Kamali M. (2014). Treatment of pistachio trees with zinc and copper in time of swollen bud in two consecutive years. *Commun. Soil Sci. Plant Anal.* 45 1025–1036. 10.1080/00103624.2014.883627

[B93] Soto S. M. Jimenez de Anta M. T. Vila J. (2006). Quinolones induce partial or total loss of pathogenicity islands in uropathogenic *Escherichia coli* by SOS-dependent or -independent pathways, respectively. *Antimicrob. Agents Chemother.* 50 649–653. 10.1128/aac.50.2.649-653.2006 16436722 PMC1366871

[B94] Stamler R. A. Heerema R. Randall J. J. (2015a). First report of phytopathogenic *Rhodococcus* isolates on pistachio bushy top syndrome ‘UCB-1’ rootstock in New Mexico. *Plant Dis.* 99 1854–1855. 10.1094/PDIS-04-15-0471-PDN 30695969

[B95] Stamler R. A. Kilcrease J. Kallsen C. Fichtner E. J. Cooke P. Heerema R. J. (2015b). First report of *Rhodococcus* isolates causing pistachio bushy top syndrome on ‘UCB-1’ rootstock in California and Arizona. *Plant Dis.* 99 1468–1476. 10.1094/pdis-12-14-1340-re 30695969

[B96] Stamler R. A. Vereecke D. Zhang Y. Schilkey F. Devitt N. Randall J. J. (2016). Complete genome and plasmid sequences for *Rhodococcus fascians* D188 and draft sequences for *Rhodococcus* isolates PBTS 1 and PBTS 2. *Genome Announc.* 4:e00495-16. 10.1128/genomeA.00495-416 27284129 PMC4901220

[B97] Stange R. R. Jeffares D. Young C. Scott D. B. Eason J. R. Jameson P. E. (1996). PCR amplification of the fas-1 gene for the detection of virulent strains of *Rhodococcus fascians*. *Plant Pathol.* 45 407–417. 10.1046/j.1365-3059.1996.d01-154.x

[B98] Stes E. Biondi S. Holsters M. Vereecke D. (2011a). Bacterial and plant signal integration via D3-type cyclins enhances symptom development in the *Arabidopsis*-*Rhodococcus fascians* interaction. *Plant Physiol.* 156 712–725. 10.1104/pp.110.171561 21459976 PMC3177270

[B99] Stes E. Vandeputte O. M. El Jaziri M. Holsters M. Vereecke D. (2011b). A successful bacterial coup d’état: how *Rhodococcus fascians* redirects plant development. *Annu. Rev. Phytopathol.* 49 69–86. 10.1146/annurev-phyto-072910-095217 21495844

[B100] Stes E. Depuydt S. De Keyser A. Matthys C. Audenaert K. Yoneyama K. (2015). Strigolactones as an auxiliary hormonal defence mechanism against leafy gall syndrome in *Arabidopsis thaliana*. *J. Exp. Bot.* 66 5123–5134. 10.1093/jxb/erv309 26136271 PMC4513927

[B101] Stes E. Francis I. Pertry I. Dolzblasz A. Depuydt S. Vereecke D. (2013). The leafy gall syndrome induced by *Rhodococcus fascians*. *FEMS Microbiol. Lett.* 342 187–194. 10.1111/1574-6968 23480693

[B102] Stes E. Prinsen E. Holsters M. Vereecke D. (2012). Plant-derived auxin plays an accessory role in symptom development upon *Rhodococcus fascians* infection. *Plant J.* 70 513–527. 10.1111/j.1365-313X.2011.04890.x 22181713

[B103] Swanson B. T. Wilkins H. F. Kennedy B. W. (1979). Factors affecting ethylene production by some plant pathogenic bacteria. *Plant Soil* 51 19–26. 10.1007/bf02205923

[B104] Temmerman W. Vereecke D. Dreesen R. Van Montagu M. Holsters M. Goethals K. (2000). Leafy gall formation is controlled by fasR, an AraC-type regulatory gene in *Rhodococcus fascians*. *J. Bacteriol.* 182 5832–5840. 10.1128/jb.182.20.5832-5840.2000 11004184 PMC94707

[B105] Teniola O. D. Addo P. A. Brost I. M. Färber P. Jany K.-D. Alberts J. F. (2005). Degradation of aflatoxin B1 by cell-free extracts of *Rhodococcus erythropolis* and *Mycobacterium fluoranthenivorans* sp. nov. DSM44556T. *Int. J. Food Microbiol.* 105 111–117. 10.1016/j.ijfoodmicro.2005.05.004 16061299

[B106] Traw M. B. Kniskern J. M. Bergelson J. (2007). SAR increases fitness of *Arabidopsis thaliana* in the presence of natural bacterial pathogens. *Evolution* 61 2444–2449. 10.1111/j.1558-5646.2007.00211.x 17725640

[B107] Vandeputte O. Öden S. Mol A. Vereecke D. Goethals K. El Jaziri M. (2005). Biosynthesis of auxin by the Gram-positive phytopathogen *Rhodococcus fascians* is controlled by compounds specific to infected plant tissues. *Appl. Environ. Microbiol.* 71 1169–1177. 10.1128/AEM.71.3.1169-1177.2005 15746315 PMC1065166

[B108] Vereecke D. (2018). Comment on “Evolutionary transitions between beneficial and phytopathogenic *Rhodococcus* challenge disease management”. *eLife* 7:e35238. 10.7554/eLife.35238 29737966 PMC5951678

[B109] Vereecke D. Burssens S. Simón-Mateo C. Inzé D. Van Montagu M. Goethals K. (2000). The *Rhodococcus fascians*-plant interaction: morphological traits and biotechnological applications. *Planta* 210 241–251. 10.1007/PL00008131 10664130

[B110] Vereecke D. Cornelis K. Temmerman W. Jaziri M. Van Montagu M. Holsters M. (2002). Chromosomal locus that affects the pathogenicity of *Rhodococcus fascians*. *J. Bacteriol.* 184 1112–1120. 10.1128/jb.184.4.1112-1120.2002 11807072 PMC134788

[B111] Vergidis P. Ariza-Heredia E. J. Nellore A. Kotton C. N. Kaul D. R. Morris M. I. (2017). *Rhodococcus* infection in solid organ and hematopoietic stem cell transplant recipients. *Emerg. Infect. Dis.* 23 510–512. 10.3201/eid2303.160633 28221102 PMC5382763

[B112] von Bargen K. Haas A. (2009). Molecular and infection biology of the horse pathogen *Rhodococcus equi*. *FEMS Microbiol. Rev.* 33 870–891. 10.1111/j.1574-6976.2009.00181.x 19453748

[B113] Wang J.-Y. Zhou L. Chen B. Sun S. Zhang W. Li M. (2015). A functional 4-hydroxybenzoate degradation pathway in the phytopathogen *Xanthomonas campestris* is required for full pathogenicity. *Sci. Rep.* 5:18456. 10.1038/srep18456 26672484 PMC4682078

[B114] Weingart H. Ullrich H. Geider K. Völksch B. (2001). The role of ethylene production in virulence of *Pseudomonas syringae* pvs. *glycinea* and *phaseolicola*. *Phytopathology* 91 511–518. 10.1094/phyto.2001.91.5.511 18943596

[B115] Wilson M. Lindow S. E. (1994). Coexistence among epiphytic bacterial-populations mediated through nutritional resource partitioning. *Appl. Environ. Microbiol.* 60 4468–4477. 16349462 10.1128/aem.60.12.4468-4477.1994 PMC202007

[B116] Wright H. Stewart J. P. Ireri R. G. Campbell I. Pow I. Reid H. W. (2003). Genome re-arrangements associated with loss of pathogenicity of the γ-herpesvirus alcelaphine herpesvirus-1. *Res. Vet. Sci.* 75 163–168. 10.1016/s0034-5288(03)00043-42 12893166

[B117] Xiao Z. Xu P. (2007). Acetoin metabolism in bacteria. *Crit. Rev. Microbiol.* 33 127–140. 10.1080/10408410701364604 17558661

[B118] Yi H.-S. Ahn Y.-R. Song G. C. Ghim S.-Y. Lee S. Lee G. (2016). Impact of a bacterial volatile 2,3-butanediol on Bacillus subtilis rhizosphere robustness. *Front. Microbiol.* 7:993. 10.3389/fmicb.2016.00993 27446033 PMC4923110

[B119] Zhou Y.-J. Zhao D.-D. Liu H. Chen H.-T. Li J.-J. Mu X.-Q. (2017). Cancer killers in the human gut microbiota: diverse phylogeny and broad spectra. *Oncotarget* 8 49574–49591. 10.18632/oncotarget.17319 28484095 PMC5564789

