# Supplementary material for: Functional Genomics Insights Into the Pathogenicity, Habitat Fitness, and Mechanisms Modifying Plant Development of Rhodococcus sp. PBTS1 and PBTS2
Source: Front Microbiol. 2020 Jan 30;11:14. doi: 10.3389/fmicb.2020.00014 (PMC7002392; doi:10.3389/fmicb.2020.00014)

Supplementary Material

**Supplementary Figure S1.** Height of control and PBTS1 and PBTS2 co-infected ‘UCB-1’ clonal plants monitored over a growth period of 4.5 months in a greenhouse. The data were first checked for normality of residuals per treatment (Shapiro-Wilk test) and homoscedasticity of variances. These assumptions were met and subsequent analysis was done using one-way analysis of variance (ANOVA), followed by a post hoc Tukey test to compare control and infected plants. No statistical differences were detected between both plant groups (p<0.05; n=10 for controls; n=25 for infected plants).

**
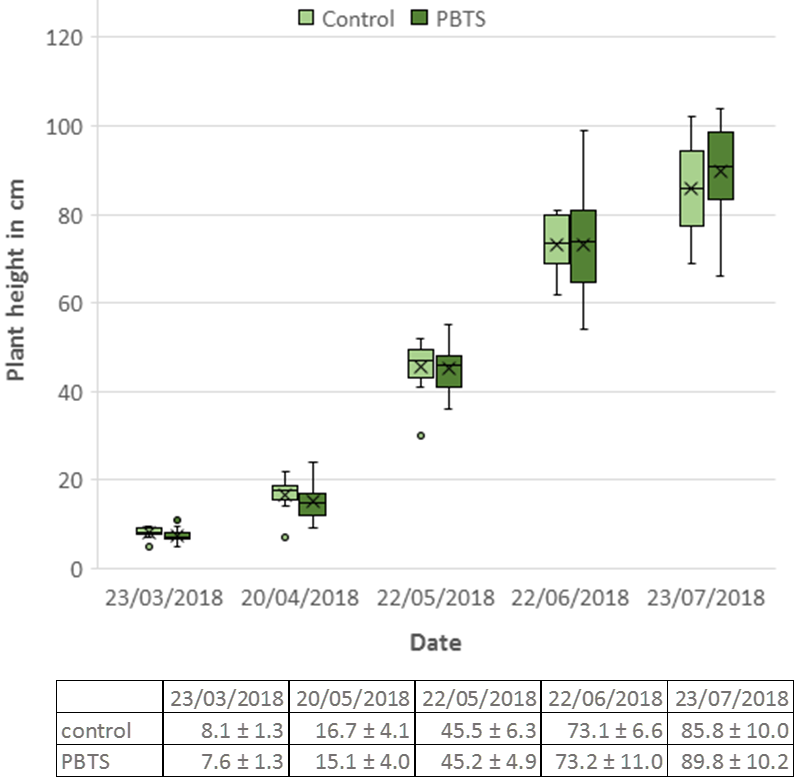
**

**Supplementary Figure S2.** Alignment of the *vic*, *fasC*, *fasD*, *fasE*, and *fasF* sequences obtained via (q)PCR from PBTS1, PBTS2, and D188 generated by Clustal Omega.

**(A)** Alignment of the *vicA* qPCR products generated with the *vicA*-EN primers on gDNA of PBTS1 and PBTS2 with *vicA* of D188 (NZ_CP015235)

*vicA*_PBTS1 ---------------------------------------------------------ATC 3

*vicA*_D188 GTCGATCTGGATCTCGAAGTGCAAACCGTTGTGCACGAACAAGATTGCCGTCGGCGCATC 1500

*vicA*_PBTS2 ---------------------------------------------------------ATC 3

***

*vicA*_PBTS1 GGGTGTGCCCTGGTATCCCAACAGCTGTGCCGGATCCTTCAGGCCCACGGTGCTGCCATC 63

*vicA*_D188 GGGTGTGCCCTGGTAGCCCAACAGCTGGGACGGATCCTTCAGGCCGACGGTGCTGCCGTC 1560

*vicA*_PBTS2 GGGTGTGCCCTGGTAGCCCACCAGCTGGGACGGATCCTTCAGGCCGACGGTGCTGCCGTC 63

*************** **** ****** * *************** *********** **

*vicA*_PBTS1 GGCCAACGTCACTGTCAGCGACGCGGCGTCGACGACGTATCCGGTGGTCCCGACGTGCGA 123

*vicA*_D188 GGCCAACGTCACCGTCAGCGAGGCGGCGTCGACGACGTATCCGGTGGTCCCGACGTGCGA 1620

*vicA*_PBTS2 GGCCAACGTCACCGTCAGCGAGGCGGCGTCGACGACGTATCCGGTGGTCCCCACGTGCGA 123

************ ******** ***************************** ********

*vicA*_PBTS1 GCCGGAGCTCAGCGGCAATGCCTCGTCGAGGAAGTCACGCGCGAACGCGATGACCTTGTC 183

*vicA*_D188 GCCGGAGCTCAGCGGCAATGCCTCGTCCAGGAAGTCACGCGCGAACGCGATGACCTTGTC 1680

*vicA*_PBTS2 GCCGGAGCTCAGCGGCAATGCCTCGTCGAGGAAGTCACGCGCGAACGCAATGACTTTGTC 183

*************************** ******************** ***** *****

*vicA*_PBTS1 TCCGCGGACCTTGTTGTAGCTCGAGCCCTTCTCGGCTCCGTCGGTCTCGGGAATGGCGTC 243

*vicA*_D188 TCCGCGGACCTTGTTGTAGCTCGTGCCCTTCTCGGCTCCGTCGGTCTCGGGGATGGCGTC 1740

*vicA*_PBTS2 TCCGCGGACCTTGTTGTAGCTCGTGCCCTTCTCGGCTCCGTCGGTCTCGGGGATGGCGTC 243

*********************** *************************** ********

*vicA*_PBTS1 CGTTCCGTAGAGGGCGTCGTAGAGCGAGCCCCATCGTGCGTTGGAGGCATTGATCGCAAA 303

*vicA*_D188 CGTTCCATAGAGGGCGTCGTAGAGCGAGCCCCATCGTGCGTTGGAGGCATTGATCGCAAA 1800

*vicA*_PBTS2 CGTTCCGTAGAGGGCGTCGTAGAGCGAGCCCCATCGTGCGTTGGAGGCATTGATCGCGAA 303

****** ************************************************** **

*vicA*_PBTS1 GCGTGCATTGAGCACAGGAACCACCAGCTGCGGCCCGGCCGTGGTGGTGATCTCGGTGTC 363

*vicA*_D188 GCGTGCATTGAGCACGGGAACCACGAGCTGCGGCCCGGCCGTGGTGGTGATCTCGGTGTC 1860

*vicA*_PBTS2 GCGTGCGTTGAGCACGGGAACCACCAGCTGCGGCCCGGCCGTGGTGGTGATCTCGGTGTC 363

****** ******** ******** ***********************************

*vicA*_PBTS1 CACGCCGGAGGTGGTGATCTGGAAGTCGGCAGGCTCGTCGAGCAGGTAGCCGATCTCTTT 423

*vicA*_D188 CACGCCGGACGTGGTGATCTGGAAGTCGGCGGGCTCGTCGAGCAGGTAGCCGATCTCTTT 1920

*vicA*_PBTS2 CACGCCGGACGTGGTGATCTGGAAGTCGGCGGGCTCGTCGAGCAGGTAGCCGATCTCTTT 423

********* ******************** *****************************

*vicA*_PBTS1 CAG--------------------------------------------------------- 426

*vicA*_D188 CAGGAAGGCCTTGTACGCTGCGCGGTCGTATTCGGCACCGGCATGCTCGCCGTGCCACGC 1980

*vicA*_PBTS2 CAG--------------------------------------------------------- 426

***

**(B)** Alignment of the *vicA2* qPCR product generated with the *vicA2*-JR primers on gDNA of PBTS1 with the published *vicA2* sequence of PBTS1 (NZ_CP015219)

*vicA2* ACCACCTCCGGGGTGGACCGGGAGATCACCTCCACCGCGGGTCCGCAGTTGGTGGTGCCG 360

*vicA2*_PBTS1 --------------------------CACCTCCACCGCGGGTCCGCAGTTGGTGGTGCCG 34

**********************************

*vicA2* GTGCTCAACGCCCGCTTCGCGATCAACGCCTCCAACGCTCGCTGGGGCTCGCTCTACGAC 420

*vicA2*_PBTS1 GTGCTCAACGCCCGCTTCGCGATCAACGCCTCCAACGCTCGCTGGGGCTCGCTCTACGAC 94

************************************************************

*vicA2* GCCCTGTACGGCACGGACGCCATCACCGAGGACGGGGGCGCCGAGAAGGGCACCTCCTAC 480

*vicA2*_PBTS1 GCCCTGTACGGCACGGACGCCATCACCGAGGACGGGGGCGCCGAGAAGGGCACCTCCTAC 154

************************************************************

*vicA2* AACCGGGTCCGCGGCGACAAGGTCATCGCCTACGCGCGGGCGTTCCTCGACGAGGCCGTG 540

*vicA2*_PBTS1 AACCGGGTCCGCGGCGACAAGGTCATCGCCTACGCGCGGGCGTTCCTCGACGAGGCCGTG 214

************************************************************

*vicA2* CCGCTGGAGTCGGGCTCGCACGTCGACGCCACGTCCTACGTGATCGACGGCTCCGCCCTC 600

*vicA2*_PBTS1 CCGCTGGAGTCGGGCTCGCACGTCGACGCCACGTCCTACGTGATCGACGGCTCCGCCCTC 274

************************************************************

*vicA2* GTGGTCACCCTCGCCTCGGGTGAGACCACCGGCCTGGCCGACCCCGCCCTGCTCGTCGGC 660

*vicA2*_PBTS1 GTGGTCACCCTCGCCTCGGGTGAGACCACCGGCCTGGCCGACCCCGCCCTGCTCGTCGGC 334

************************************************************

*vicA2* TACACCGGCGAGCCGTCGGCGCCCACGGGCATCCTGTTCCGCCACCACGGCCTGCACCTC 720

*vicA2*_PBTS1 TACACCGGCGAGCCGTCGGCGCCCACGGGCATCCTGTTCCGCCACCACA----------- 383

************************************************

**(C)** Alignment of the *fasD* PCR products generated with the *fasD*_DV primers and qPCR products generated with the *fasD*-JR primers on gDNA of PBTS1 and PBTS2 with *fasD* (*pFi_080*; JN093097) of pFiD188

*pFi_080* ATGAAGGAATCAACCATGGCACAGACGCAAGCAAGGTTTGATCGGGTGCGATGGGAACCC 60

PBTS1_qPCR ------------------------------------------------------------ 0

PBTS2_qPCR ------------------------------------------------------------ 0

PBTS1_PCR ----------------------------------GGTTTGATCGGGTGCGATGGGAACCC 26

PBTS2_PCR ----------------------------------GGTTTGATCGGGTGCGATGGGAACCC 26

*pFi_080* GGCGTATACGCAATCGTCGGTGCCACCGGAATTGGAAAGAGCGCCGAAGCGAGCAAGTTG 120

PBTS1_qPCR ------------------------------------------------------------ 0

PBTS2_qPCR ------------------------------------------------------------ 0

PBTS1_PCR GGCGTATACGCAATCGTCGGTGCCACCGGAATTGGAAAGAGCGCCGAAGCGAGCAAGTTG 86

PBTS2_PCR GGCGTATACGCAATCGTCGGTGCCACCGGAATTGGAAAGAGCGCCGAAGCGAGCAAGTTG 86

*pFi_080* GCATTGAGTCACTCGGCTCCGATTGTTGTTGCCGACCGTATCCAGTGTTACTCCGATCTC 180

PBTS1_qPCR ------------------------------------------------------------ 0

PBTS2_qPCR ------------------------------------------------------------ 0

PBTS1_PCR GCATTGAGTCACTCGGCTCCGATTGTTGTTGCCGACCGTATCCAGTGTTACTCCGATCTC 146

PBTS2_PCR GCATTGAGTCACTCGGCTCCGATTGTTGTTGCCGACCGTATCCAGTGTTACTCCGATCTC 146

*pFi_080* CTGGTCACCAGTGGTCGAGCGTTCGACGCGAAAGTGGAAGGGCTCAACCGCGTTTGGCTC 240

PBTS1_qPCR ------------------------------------------------------------ 0

PBTS2_qPCR ------------------------------------------------------------ 0

PBTS1_PCR CTGGTCACCAGTGGTCGAGCGTTCGACGCGAAAGTGGAAGGGCTCAACCGCGTTTGGCTC 206

PBTS2_PCR CTGGTCACCAGTGGTCGAGCGTTCGACGCGAAAGTGGAAGGGCTCAACCGCGTTTGGCTC 206

*pFi_080* GACAACCGGACCATACATCAGGGCAACTTCGATCCGGACGAGGCCTTTGACCGGCTGATA 300

PBTS1_qPCR ------------------------------------------------------------ 0

PBTS2_qPCR ------------------------------------------------------------ 0

PBTS1_PCR GACAACCGGACCATACATCAGGGCAACTTCGATCCGGACGAGGCCTTTGACCGGCTGATA 266

PBTS2_PCR GACAACCGGACCATACATCAGGGCAACTTCGATCCGGACGAGGCCTTTGACCGGCTGATA 266

*pFi_080* AAAGTACTGACCTCGTACGTTGATCGCGGCGAAGCGGTGGTAATGGAGGGCGGTTCGATA 360

PBTS1_qPCR ------------------------------------------------------------ 0

PBTS2_qPCR ------------------------------------------------------------ 0

PBTS1_PCR AAAGTACTGACCTCGTACGTTGATCGCGGCGAAGCGGTGGTAATGGAGGGCGGTTCGATA 326

PBTS2_PCR AAAGTACTGACCTCGTACGTTGATCGCGGCGAAGCGGTGGTAATGGAGGGCGGTTCGATA 326

*pFi_080* TCGCTCATTCTTCGATTCGCGCAAACGATATCCAACCTACCGTTCCCAGCTGTCGTGAAT 420

PBTS1_qPCR ------------------------------------------------------------ 0

PBTS2_qPCR ------------------------------------------------------------ 0

PBTS1_PCR TCGCTCATTCTTCGATTCGCGCAAACGATATCCAACCTACCGTTCCCAGCTGTCGTGAAT 386

PBTS2_PCR TCGCTCATTCTTCGATTCGCGCAAACGATATCCAACCTACCGTTCCCAGCTGTCGTGAAT 386

*pFi_080* GTTATGCCTATTCCTGATAGGCAACACTACTTTGCCCAGCAGTGCGCAAGGGCACGACAA 480

PBTS1_qPCR ---------------------CAACACTACTTTGCCCAGCAGTGCGCAAGGGCACGACAA 39

PBTS2_qPCR ---------------------CAACACTACTTTGCCCAGCAGTGCGCAAGGGCACGACAA 39

PBTS1_PCR GTTATGCCTATTCCTGATAGGCAACACTACTTTGCCCAGCAGTGCGCAAGGGCACGACAA 446

PBTS2_PCR GTTATGCCTATTCCTGATAGGCAACACTACTTTGCCCAGCAGTGCGCAAGGGCACGACAA 446

***************************************

*pFi_080* ATGTTACGGGGAGATTCAACGGGCAGGAATTTACTCACCGAACTGGCGGAAGCGTGGGTT 540

PBTS1_qPCR ATGTTACGGGGAGATTCAACGGGCAGGAATTTACTCACCGAACTGGCGGAAGCGTGGGTT 99

PBTS2_qPCR ATGTTACGGGGAGATTCAACGGGCAGGAATTTACTCACCGAACTGGCGGAAGCGTGGGTT 99

PBTS1_PCR ATGTTACGGGGAGATTCAACGGGCAGGAATTTACTCACCGAACTGGCGGAAGCGTGGGTT 506

PBTS2_PCR ATGTTACGGGGAGATTCAACGGGCAGGAATTTACTCACCGAACTGGCGGAAGCGTGGGTT 506

************************************************************

*pFi_080* CTAGGCGATCAGCACAACTTCATAGCCTCGGTTGCGGGTCTGGACTGTGTACTCGACTGG 600

PBTS1_qPCR CTAGGCGATCAGCACAACTTCATAGCCTCGGTTGCGGGTCTGGACTGTGTACTCGACTGG 159

PBTS2_qPCR CTAGGCGATCAGCACAACTTCATAGCCTCGGTTGCGGGTCTGGACTGTGTACTCGACTGG 159

PBTS1_PCR CTAGGCGATCAGCACAACTTCATAGCCTCGGTTGCGGGTCTGGACTGTGTACTCGACTGG 566

PBTS2_PCR CTAGGCGATCAGCACAACTTCATAGCCTCGGTTGCGGGTCTGGACTGTGTACTCGACTGG 566

************************************************************

*pFi_080* TGCGCCACGCATTCCGTAACACCAGAGGAGTTGGCCAACCGAGACCTCACCACGGAGGTG 660

PBTS1_qPCR TGCGCCACGCATTCCGTAACACCAGAGGAGTTGGCC------------------------ 195

PBTS2_qPCR TGCGCCACGCATTCCGTAACACCAGAGGAGTTGGCC------------------------ 195

PBTS1_PCR TGCGCCACGCATTCCGTAACACCAGAGAAGTTTGGC------------------------ 602

PBTS2_PCR TGCGCCACGCATTCCGTAACACCAGAGAAAGTTGGC------------------------ 602

*************************** * * * *

**(D)** Alignment of the *fasC* qPCR products generated with the *fasD*-PJ primers on gDNA of PBTS1 and PBTS2 with *fasC* (*pFi_079*; JN093097) of pFiD188

*fasC*_PBTS1 -------------------------------------------CGGTTGAGTTCGCCTTC 17

*fasC*_D188 GATGTCGGCGCAGTCCTGGTCAGTTGGGGAGCCTCCTACGACTTGTCGGAGTTCGCCTTC 180

*fasC*_PBTS2 ------------------------------------------------GAGTTCGCCTTC 12

************

*fasC*_PBTS1 TCCCATTTCACCCCGGGCGATATCACCGTCATCGATTCGATGCCCAACTGGACAGTTCAC 77

*fasC*_D188 TCCCATTTCACCCCGGGCGATATCACCGTCATCGATTCGATGCCCAACTGGACAGTTCAC 240

*fasC*_PBTS2 TCCCATTTCACCCCGGGCGATATCACCGTCATCGATTCGATGCCCAACTGGACAGTTCAC 72

************************************************************

*fasC*_PBTS1 GTGCCAGGCCATCCACAGGAGGCCGCCGATCTTCTCCTCGAATCGCTACCAGGCGATGGA 137

*fasC*_D188 GTGCCAGGCCATCCACAGGAGGCCGCCGATCTTCTCCTCGAATCGCTACCAGGCGATGGA 300

*fasC*_PBTS2 GTGCCAGGCCATCCACAGGAGGCCGCCGATCTTCTCCTCGAATCGCTACCAGGCGATGGA 132

************************************************************

*fasC*_PBTS1 CGGGTCTACCTACGGCTGTCGAGTCAGGTCAACCGTTACCCCCATGCGGTCCGAGGGACA 197

*fasC*_D188 CGGGTCTACCTACGGCTGTCGAGTCAGGTCAACCGTTACCCCCATGCGGTCCGAGGGACA 360

*fasC*_PBTS2 CGGGTCTACCTACGGCTGTCGAGTCAGGTCAACCGTTACCCCCATGCGGTCCGAGGGACA 192

************************************************************

*fasC*_PBTS1 TCGTTCACACCTATCAAGTACGGCACCCGCGGAGTCGTCCTCGCAGTCGGGCCCTGTTTA 257

*fasC*_D188 TCGTTCACACCTATCAAGTACGGCACCCGCGGAGTCGTCCTCGCAGTCGGGCCCTGTTTA 420

*fasC*_PBTS2 TCGTTCACACCTATCAAGTACGGCACCCGCGGAGTCGTCCTCGCAGTCGGGCCCTGTTTA 252

************************************************************

*fasC*_PBTS1 GATGCGGTGCTGTCG--------------------------------------------- 272

*fasC*_D188 GATGCGGTGCTGTCGGCAACCTCCATGCTCGACGTAACCATCCTCTACGCGGCGACGATT 480

*fasC*_PBTS2 GATGCGGTGCTGTCG--------------------------------------------- 267

***************

**(E)** Alignment of the qPCR products generated with the *fasE*-PJ primers on gDNA of PBTS1 and PBTS2 with *fasE* (*pFi_081*; JN093097) of pFiD188

*fasE*_D188 AGTGAACTTTTCGACGCCGTGCGCGGCGGCCTCGGTCAATTCGGCGTAATCGTCAACGCG 360

*fasE*_PBTS1 ------------------------------------------------------------ 0

*fasE*_PBTS2 ------------------------------------------------------------ 0

*fasE*_D188 ACAATCCGTCTGACCGCTGCTCACGAGTCGGTTCGACAGTACAAATTGCAGTATTCCAAC 420

*fasE*_PBTS1 ACAATCCGTCTGACCGCTGCTCACGAGTCGGTTCGACAGTACAAATTGCAGTATTCCAAC 60

*fasE*_PBTS2 ACAATCCGTCTGACCGCTGCTCACGAGTCGGTTCGACAGTACAAATTGCAGTATTCCAAC 60

************************************************************

*fasE*_D188 CTCGGCGTATTCCTTGGCGACCAACTCCGCGCCATGTCCAACAGACTATTCGACCATGTA 480

*fasE*_PBTS1 CTCGGCGTATTCCTTGGCGACCAACTCCGCGCCATGTCCAACAGACTATTCGACCATGTA 120

*fasE*_PBTS2 CTCGGCGTATTCCTTGGCGACCAACTCCGCGCCATGTCCAACAGACTATTCGACCATGTA 120

************************************************************

*fasE*_D188 CAAGGACGAATTCGTGTCGATGCCGACGGCCACTTACGTTATCGACTGGACCTAGCCAAG 540

*fasE*_PBTS1 CAAGGACGAATTCGTGTCGATGCCGACGGCCACTTACGTTATCGACTGGACCTAGCCAAG 180

*fasE*_PBTS2 CAAGGACGAATTCGTGTCGATGCCGACGGCCACTTACGTTATCGACTGGACCTAGCCAAG 180

************************************************************

*fasE*_D188 TACTTCACCCCACCACGAAGGCCAGACGACGATGCGCTGTTGTCATCGCTCCAATACGAT 600

*fasE*_PBTS1 TACTTCACCCCACCACGAAGGCCAGACGACGATGCGCTGTTGTCATCGCTCCAATACGAT 240

*fasE*_PBTS2 TACTTCACCCCACCACGAAGGCCAGACGACGATGCGCTGTTGTCATCGCTCCAATACGAT 240

************************************************************

*fasE*_D188 TCGTGCGCCGAATACAACTCGGACGTAGATTATGGTGACTTTATCAACCGTATGGCGGAT 660

*fasE*_PBTS1 TCGTGCGCCGAATACAACTCGGACGTAGATTATGGTGACTTTATCAACCGTATGGCGGAT 300

*fasE*_PBTS2 TCGTGCGCCGAATACAACTCGGACGTAGATTATGGTGACTTTATCAACCGTATGGCGGAT 300

************************************************************

*fasE*_D188 CAGGAGCTTGATCTTCGGCACACAGGTGAGTGGTTCTATCCACATCCATGGGCCAGCCTG 720

*fasE*_PBTS1 CAGGAGCTTGATCTTCGGCACACAGGTGAGTGGTTCTATCCACATCCATGGGCCAGCCTG 360

*fasE*_PBTS2 CAGGAGCTTGATCTTCGGCACACAGGTGAGTGGTTCTATCCACATCCATGGGCCAGCCTG 360

************************************************************

*fasE*_D188 CTGATCCCGGCAGACAAGATCGAGCAGTTCATCGAAACTACCAGCTCCTCATTGACGGAT 780

*fasE*_PBTS1 CTGATCCCGGCAGACAAGATCGAGCAGTTCATCGAAACTACCAGCTCCTCATTGACGGAT 420

*fasE*_PBTS2 CTGATCCCGGCAGACAAGATCGAGCAGTTCATCGAAACTACCAGCTCCTCATTGACGGAT 420

************************************************************

*fasE*_D188 GACCTAGGAAACTCCGGGCTGATCATGGTTTACCCGATTCCAACAACACCGATCACCGCA 840

*fasE*_PBTS1 GACCTAGGAAACTCCGGGCTGATCATGGTTTACCCGATTCCAACAACACCGATCACCGCA 480

*fasE*_PBTS2 GACCTAGGAAACTCCGGGCTGATCATGGTTTACCCGATTCCAACAACACCGATCACCGCA 480

************************************************************

*fasE*_D188 CCGTTCATTCCGATTCCTCACTGCGACACATTCTTCATGTTGGCCGTACTTCGAACAGCA 900

*fasE*_PBTS1 CCGTTCATTCCGATTCCTCACTGCGACACATTCTTCATGTTGGCCGTACTTCGAACAGCA 540

*fasE*_PBTS2 CCGTTCATTCCGATTCCTCACTGCGACACATTCTTCATGTTGGCCGTACTTCGAACAGCA 540

************************************************************

*fasE*_D188 TCCCCAGGGGCCGAAGCCCGAATGATTGCCAGCAACCGCCTGCTCTATGAACAGGCTCGG 960

*fasE*_PBTS1 TCCCCAGGGGCCGAAGCCCGAATGATTGCCAGCAACCGCCTGCTCTATGAACAGGCTCGG 600

*fasE*_PBTS2 TCCCCAGGGGCCGAAGCCCGAATGATTGCCAGCAACCGCCTGCTCTATGAACAGGCTCGG 600

************************************************************

*fasE*_D188 GATGTAGGCGGGGTGGCCTACGCAGTCAATGCTGTCCCCATGTCACCGGGAGATTGGTGT 1020

*fasE*_PBTS1 GATGTAGGCGGGGTGGCCTACGCAGTCAATGCTGTCCCCATGTCACCGGGAGATTGGTGT 660

*fasE*_PBTS2 GATGTAGGCGGGGTGGCCTACGCAGTCAATGCTGTCCCCATGTCACCGGGAGATTGGTGT 660

************************************************************

*fasE*_D188 ACGCACTTCGGTTCGCGATGGCAGGCCATCGCACGGGCTAAGCGTCGCTTCGACCCATAC 1080

*fasE*_PBTS1 ACGCACTTCG-------------------------------------------------- 670

*fasE*_PBTS2 ACGCACTTCGG------------------------------------------------- 671

**********

**(F)** Alignment of the qPCR products generated with the *fasF*-PJ primers on gDNA of PBTS1 and PBTS2 with *fasF* (*pFi_082*; JN093097) of pFiD188

*fasF*_D188 GTCGGCCTCATGGGCACCCTCGCGAACGCGGCCTTGGACTCCGGCGGAACCGTCGTTGGC 240

*fasF*_PBTS1 ----------------------------------------------GAACCGTCGTTGGC 14

*fasF*_PBTS2 ----------------------------------------------GAACCGTCGTTGGC 14

**************

*fasF*_D188 GTGATCCCTGAGAGCTTCACTGCGATACCCGAGGCTGCGCATCATGGACTGACAGAACTA 300

*fasF*_PBTS1 GTGATCCCTGAGAGCTTCACTGCGATACCCGAGGCTGCGCATCATGGACTGACAGAACTA 74

*fasF*_PBTS2 GTGATCCCTGAGAGCTTCACTGCGATACCCGAGGCTGCGCATCATGGACTGACAGAACTA 74

************************************************************

*fasF*_D188 CACGTCGTCCATGACATGCACCAACGCAAAGCTCTCATGGCCGAACTCGGTGACGCATTC 360

*fasF*_PBTS1 CACGTCGTCCATGACATGCACCAACGCAAAGCTCTCATGGCCGAACTCGGTGACGCATTC 134

*fasF*_PBTS2 CACGTCGTCCATGACATGCACCAACGCAAAGCTCTCATGGCCGAACTCGGTGACGCATTC 134

************************************************************

*fasF*_D188 ATTGCCCTCCCCGGCGGTGTCGGAACCGCAGAAGAGTTCTTCGAGGTCCTTACGTGGTCA 420

*fasF*_PBTS1 ATTGCCCTCCCCGGCGGTGTCGGAACCGCAGAAGAGTTCTTCGAGGTCCTTACGTGGTCA 194

*fasF*_PBTS2 ATTGCCCTCCCCGGCGGTGTCGGAACCGCAGAAGAGTTCTTCGAGGTCCTTACGTGGTCA 194

************************************************************

*fasF*_D188 CACCTGGGGCTTCACAATAAACCCTGTGTACTGCTGAACGACAACGAGTATTACCGCCCC 480

*fasF*_PBTS1 CACCTGGGGCTTCACAATAAACCCTGTGTACTGCTGAACGACAACGAGTATTACCGCCCC 254

*fasF*_PBTS2 CACCTGGGGCTTCACAATAAACCCTGTGTACTGCTGAACGACAACGAGTATTACCGCCCC 254

************************************************************

*fasF*_D188 TTGCTCTCCTACATCGAGCACGCTGCCGTCGAAGGATTTATCACCCCCGCAACCCGGTCT 540

*fasF*_PBTS1 TTGCTCTC---------------------------------------------------- 262

*fasF*_PBTS2 TTGCTCTC---------------------------------------------------- 262

**Supplementary Figure S3.** Pairwise ANI and *is*DDH values of the published PBTS1 and PBTS2 genome sequences, those from two newly sequenced PBTS1-like and PBTS2-like isolates, and *R. fascians* D188 and *R. corynebacterioides* NBRC 14404 as references.

The lower triangle displays ANI values (%), and the upper triangle displays *is*DDH values (%). Boxes with ANI ≥96% or *is*DDH ≥69.5% are colored red. Strains belonging to the same species are marked with the same color.

**
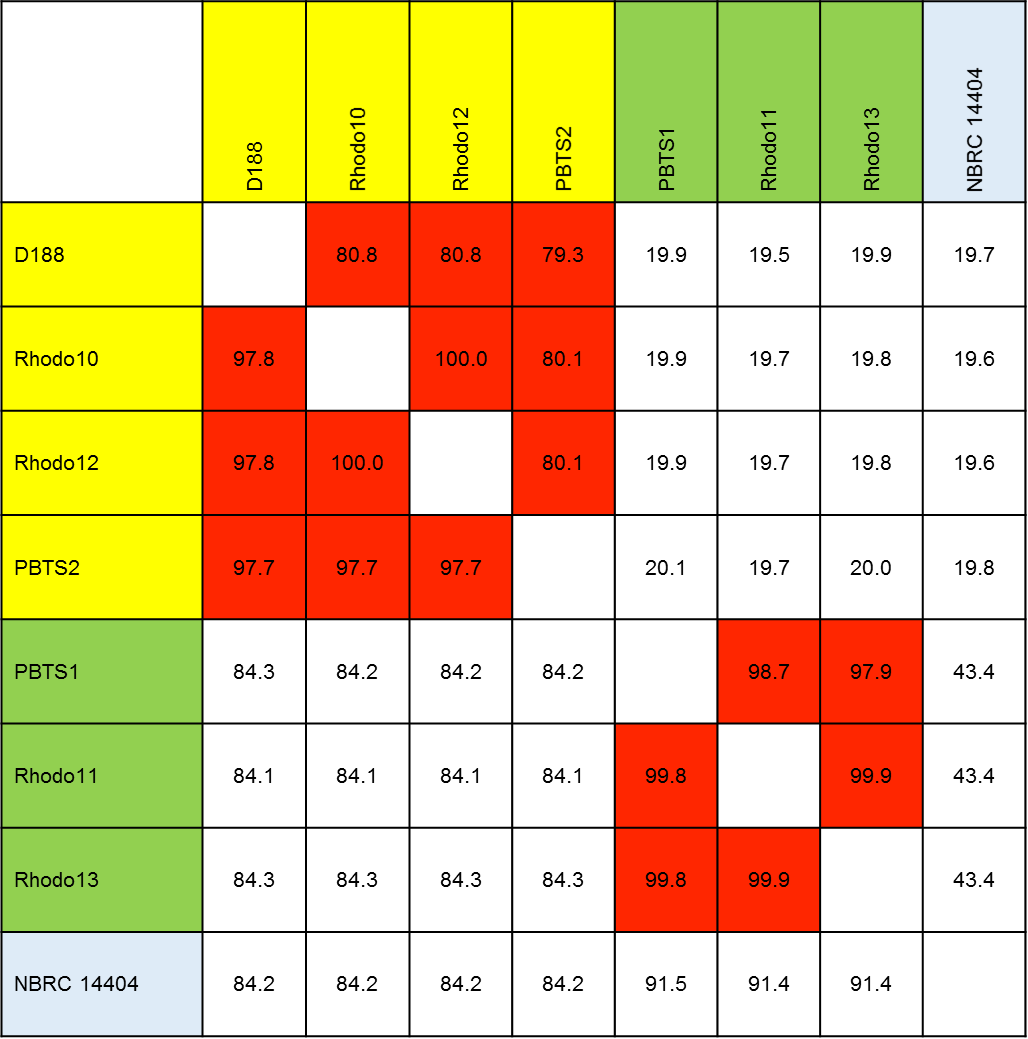
**

**Supplementary Figure S4.** Cytokinin production pathway via tRNA degradation in PBTS1, PBTS2, and D188.

Precursor production via the MEP pathway, modification of iP in the tRNA and subsequent degradation results in the production of iP, cZ, and 2MeSZ. The production of these cytokinins by PBTS1 and PBTS2 needs to be experimentally demonstrated. Gene locus tags in PBTS1 are indicated in red, those of PBTS2 in orange, and those of D188 in black.

**
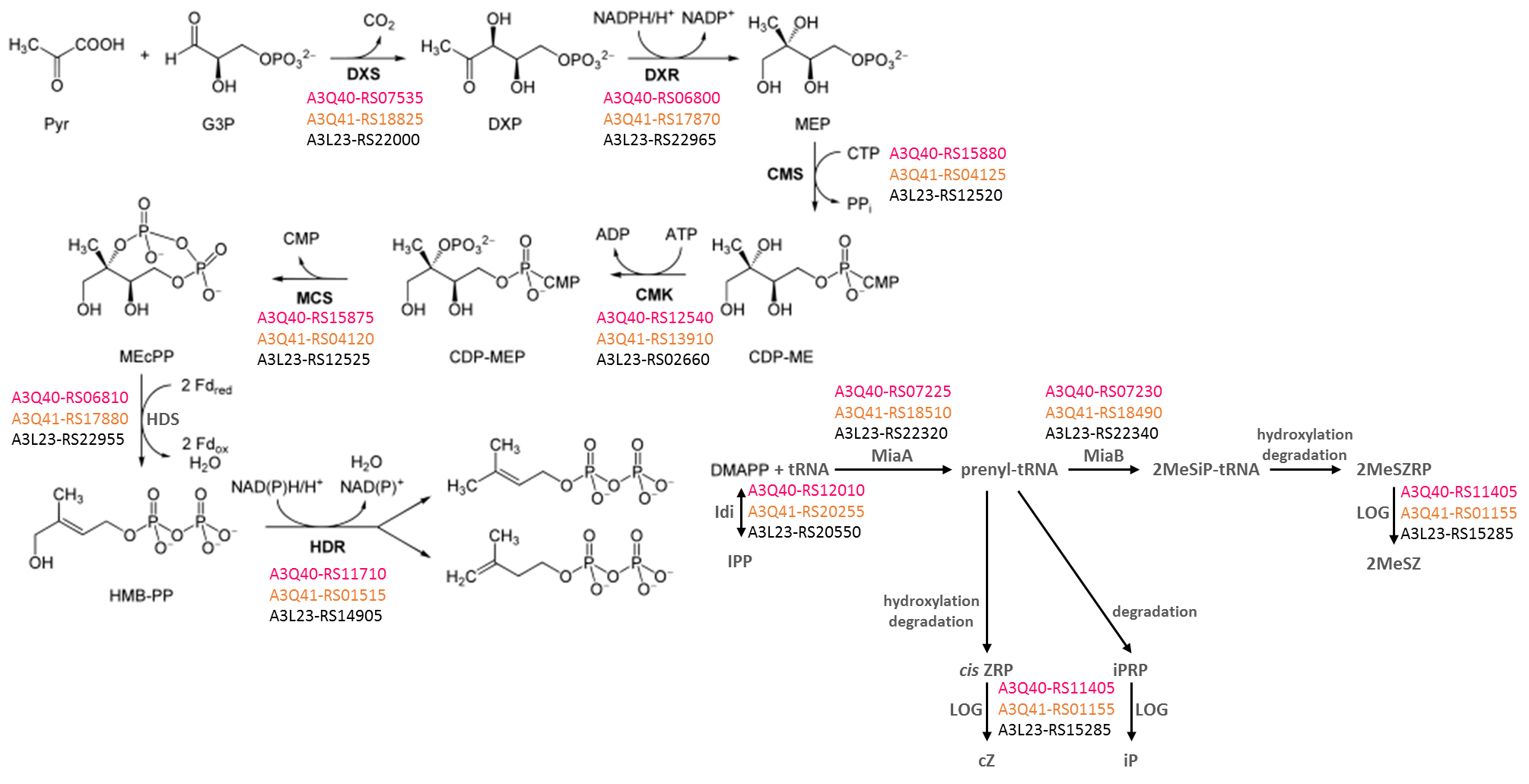
**

**Supplementary Figure S5.** Tryptophan, 2,3-butanediol, and IAA biosynthesis pathway in PBTS1, PBTS2, and D188. Gene locus tags in PBTS1 are indicated in red, those of PBTS2 in orange, and those of D188 in black.


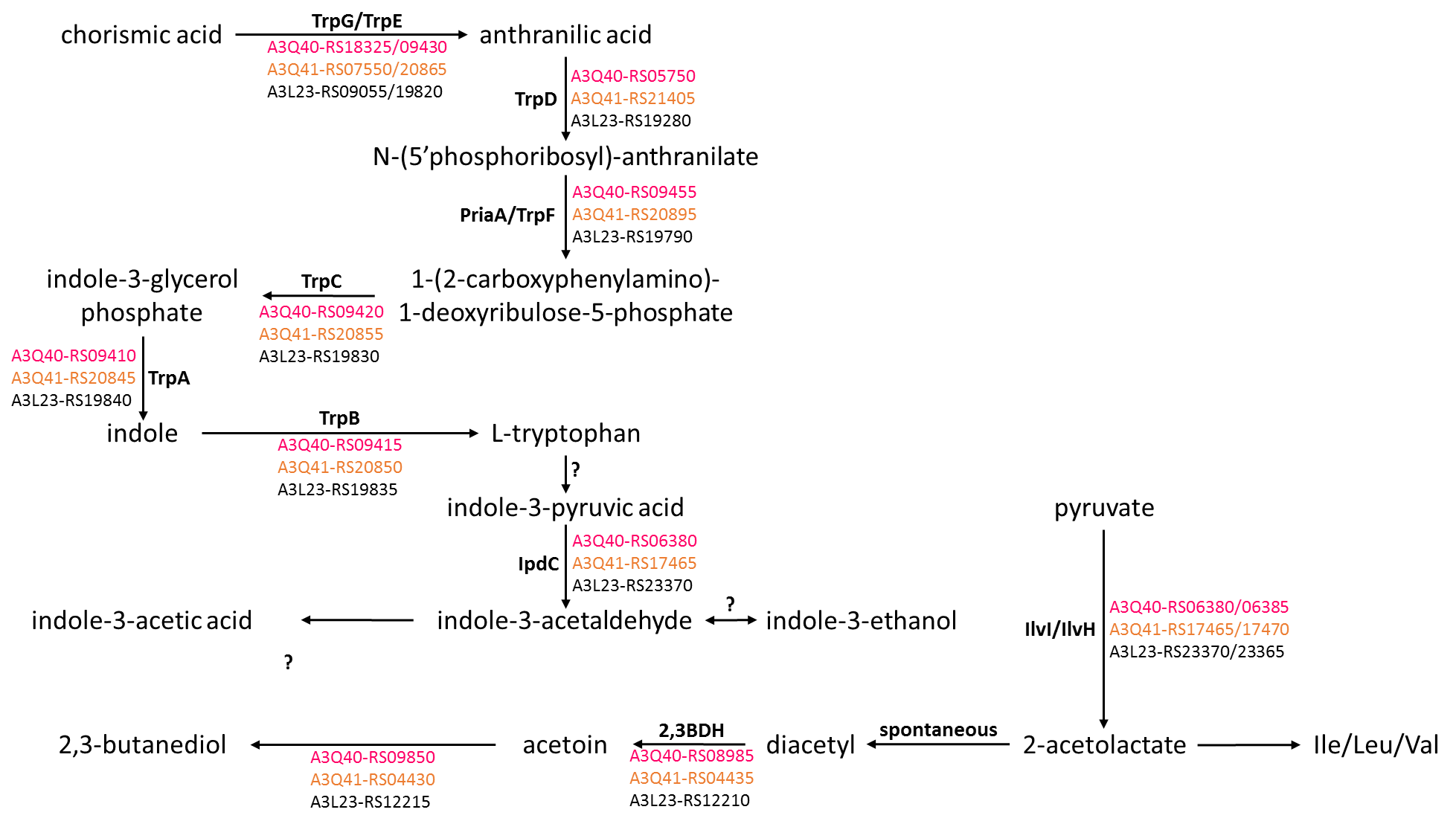

Supplement: Supplementary file 1 [file Data_Sheet_1.DOCX]
